# Analysis of xyloglucan metabolism mutants highlights the prominent role of xylose cleavage in seed dormancy

**DOI:** 10.1111/tpj.70063

**Published:** 2025-03-31

**Authors:** Hiromi Suzuki, Parisa Savane, Lucile Marion‐Poll, Julien Sechet, Anne Frey, Adeline Berger, Katia Belcram, Nero Borrega, Mitsunori Seo, Aline Voxeur, Grégory Mouille, Annie Marion‐Poll

**Affiliations:** ^1^ Université Paris‐Saclay, INRAE, AgroParisTech, Institute Jean‐Pierre Bourgin for Plant Sciences (IJPB) 78000 Versailles France; ^2^ RIKEN Center for Sustainable Resource Science Yokohama Kanagawa 230‐0045 Japan; ^3^ Department of Basic Neurosciences, Faculty of Medicine University of Geneva Geneva Switzerland; ^4^ Present address: School of Bioscience and Biotechnology Tokyo University of Technology Tokyo 192‐0982 Japan; ^5^ Present address: Alkion BioInnovations 78000 Versailles France; ^6^ Present address: Université Clermont Auvergne, INRAE, UR QuaPA 63122 Saint‐Genès Champanelle France; ^7^ Present address: Tropical Biosphere Research Center University of the Ryukyus Okinawa 903‐0213 Japan

**Keywords:** seed, dormancy, germination, xyloglucan, cell wall, xylosidase, Arabidopsis

## Abstract

Seed dormancy is an adaptive trait that delays germination until environmental conditions become favorable for seedling survival and growth. Germination has been shown to depend on the mechanical resistance strength of the covering layers (testa and endosperm) that counteracts the growth force of the embryo. Cell wall remodeling is essential in the regulation of germination processes. In *Arabidopsis thaliana*, the side chain trimming of xyloglucans (XyG), the major hemicellulose in cell walls, by the apoplastic XYLOSIDASE1 (XYL1), has been previously shown to regulate XyG side chain length and seed dormancy. To investigate to what extent side chain complexity impacts on cell wall mechanical properties and regulates seed germination, *xyl1* mutations were combined here with mutations in the two other glycosidases, the fucosidase AXY8 and the beta‐galactosidase BGAL10. Analysis of germination phenotypes in *axy8 bgal10 xyl1* and in several XyG biosynthesis mutants did not show any link between dormancy depth and side chain length. The very specific effect of *xyl1* on seed dormancy in single and multiple mutants was clearly correlated with alterations in XyG intracellular localization, together with release and oxidation of free oligosaccharides (XGO). Accumulation of oxidized XGO could negatively impact cell wall remodeling by impairing remobilization and polarized secretion in cell walls, thus reducing growth anisotropy in elongating organs and modifying mechanical characteristics in seed tissues.

## Significance Statement

Analysis of mutants differently impaired in xyloglucan (XyG) metabolism shows that XYL1 glycosidase loss‐of‐function has a specific impact on seed dormancy. Germination of xyl1 seeds is associated with the release and oxidation of free oligosaccharides (XGO) and defective XyG accumulation in cell walls, suggesting that xylose removal is essential for XGO recycling and XyG remodeling in order to fine‐tune mechanical resistance of seed tissues and dormancy maintenance.

## INTRODUCTION

Seed germination results from the coordination of the induction of embryo growth and weakening of the protective covering tissues so that radicle emergence can occur. Seed dormancy is an adaptive trait that prevents germination. It is induced during seed development and inhibits precocious germination on the mother plant. After dispersion, it enables seeds to remain quiescent until the conditions for germination and seedling establishment become favorable (Graeber et al., 2012). In *Arabidopsis thaliana*, seed dormancy is released by a period of dry storage (after‐ripening) or by various treatments, such as imbibition in the presence of exogenous gibberellin (GA) or in cold and dark conditions (stratification).

Upon imbibition, seed germination is triggered by endogenous and environmental signals that activate mechanisms underlying cell wall production and remodeling. These processes direct cell expansion for embryo axis elongation or modify cell wall properties of the endosperm for the rupture of the micropylar zone that faces the radicle (Steinbrecher & Leubner‐Metzger, 2017). The principal constituents of cell walls are crystalline cellulose microfibrils, hemicelluloses, and pectins. In Arabidopsis, as in most angiosperms, xyloglucans (XyG) are the major hemicellulose polymers in cell walls and are strongly associated with cellulose through hydrogen bonds (Cosgrove, 2022; Pauly & Keegstra, 2016). XyG have a β‐1,4‐linked glucan backbone that is substituted with xylosyl residues, which are often substituted further with galactose that can be *O*‐acetylated. Fucose can also be appended to some galactosyl residues. As in many other dicots, the xylosylation pattern in Arabidopsis is generally regular, consisting mainly of XXXG‐type units. The pattern of XyG substitutions is described using a single‐letter nomenclature (Figure S1).

XyG are synthetized by a series of glycosyltransferases (GT) in the Golgi and secreted to the cell surface where they are incorporated in the wall matrix (Julian & Zabotina, 2022). In Arabidopsis, a family of five cellulose synthase‐like C (CSLC) proteins from the GT2 family are responsible for the synthesis of the XyG glucan backbone (Kim et al., 2020). The xylosylation of the glucan backbone is catalyzed by five xyloglucan 6‐α‐d‐xylosyltransferases, AtXXT1‐5 (Zhong et al., 2021). Despite the lack of XyG, multiple *cslc* and *xxt* mutants grow normally and exhibit only mild developmental phenotypes, suggesting that compensatory mechanisms may occur, such as the observed increase in homogalacturonan and glucomannan, as recently reported (Bou Daher et al., 2024; Sowinski et al., 2022).

Two galactosyltransferases MURUS 3 (MUR3) and XYLOGLUCAN L‐SIDE CHAIN GALACTOSYLTRANSFERASE POSITION 2 (XLT2) from the GT47 family are involved in XyG galactosylation, respectively, at the third and the second xylosyl residues of XXXG subunits to form XLXG, XXLG, and XLLG (Jensen et al., 2012; Madson et al., 2003). Two *O*‐acetyl transferases of the Trichome Birefringence‐Like protein family, AXY4 and AXY4‐LIKE (AXY4L), specifically act on XyG and exclusively attach the *O*‐acetyl substituent to galactosyl residues, which can also carry a fucosyl residue (Gille et al., 2011). In *axy4* mutants, an absence of *O*‐acetylation is observed in vegetative tissues and does not affect growth. In *axy4L*, only seeds lack acetyl residues. Since MUR2, a fucosyltransferase from the GT37 family, is only active on the second galactosyl residue of XLLG, extension with a fucosyl residue occurs at the third side chain. XLFG is the most complex XyG subunit in Arabidopsis (Vanzin et al., 2002). While *mur2* and *xlt2* mutants grow normally, loss‐of‐function *mur3* alleles exhibit severe vegetative phenotypes and multiple cellular defects. The inability to extend the side chains and the presence of large tandemly repeated XXXG domains were reported to be major factors in the XyG dysfunctional properties that prevent normal growth of the loss‐of‐function *mur3* alleles (Kong et al., 2015). More recently, the presence of intracellular aggregations in *mur3* has been attributed to defective secretion of low‐substituted XyG (Hoffmann & McFarlane, 2024).

Fucose is provided in the form of GDP‐fucose which is produced from GDP‐mannose by a bifunctional GDP‐4‐keto‐6‐deoxymannose‐3,5‐epimerase‐4‐reductase (GER) which acts downstream a GDP‐mannose‐4,6‐dehydratase (GMD). The mutation of *MUR1* which encodes an isoform of GMD results in XyG almost devoid of fucosyl residues. However, the vegetative phenotypes of *mur1* mutants have been reported to result from a concomitant defect in the structure of the pectic polysaccharide rhamnogalacturonan II (RGII), rather than from altered XyG (Bonin et al., 2003; Bonin & Reiter, 2000).

After synthesis in the Golgi, XyG are exported to the apoplast via vesicle secretion and can undergo structural maturation and turnover. Apoplastic modifying enzymes include endotransglucosylase/endohydrolases (XTH) that cleave and religate XyG chains and thus contribute to cell wall remodeling during cell elongation (Pauly & Keegstra, 2016). Several glycosidases can degrade XyG by trimming side chains, such as α‐xylosidase 1 (XYL1), β‐galactosidase 10 (BGAL10), and α‐fucosidase ALTERED XYLOGLUCAN 8 (AXY8) (Günl et al., 2011; Sampedro et al., 2001, 2012). Loss‐of‐function of these glycosidases results in significant modifications in XyG composition, however mutants display only minor phenotypes. The most obvious alterations are observed in *xyl1* mutants lacking α‐xylosidase activity, which exhibit various phenotypic defects such as reduced silique elongation, increased cell stiffness and overaccumulation of free XyG oligosaccharides (XGO) (Di Marzo et al., 2022; Sampedro et al., 2010; Sechet et al., 2016; Shigeyama et al., 2016). In seeds, the *xyl1* mutation has been shown to reduce dormancy, increase germination resistance to both thermoinhibition and paclobutrazol, a GA biosynthesis inhibitor (Günl & Pauly, 2011; Sechet et al., 2016; Shigeyama et al., 2016). Germination phenotypes have been shown to be associated with only slight modifications in ABA and GA contents and differential expression of genes encoding specific ABA and GA metabolism enzymes. It has been suggested that altered cell wall properties indirectly affected seed germination through the regulation of hormone gene expression (Shigeyama et al., 2016). Little is known about the regulation of these apoplastic glycosidases, nevertheless a recent study provided molecular and genetic evidence of the regulation of *XYL1* expression by the transcription factor SEEDSTICK (Di Marzo et al., 2022).

How variations in side chains influence XyG physiological functions is still poorly understood. In *xyl1* mutant seeds, a reduction in the relative abundance of fucosylated subunits has been observed in both embryo and testa/endosperm (TE) fractions (Sechet et al., 2016), as also reported in vegetative tissues (Günl et al., 2011; Sampedro et al., 2010). In germinating *xyl1* embryo, reduced side chain length was associated with lower XyG accumulation in cell walls and higher accumulation in intracellular vesicles, suggesting defects in XyG remobilization or secretion. Furthermore, restoration of normal XyG composition in endosperm tissues was associated with restoration of dormancy in transgenic seeds, indicating that endosperm mechanical resistance could be reduced in mutant seeds and could not counteract embryo growth (Sechet et al., 2016). Recently, addition of d‐galacturonic acid onto XyG in *mur3* mutants, whose side chains lack L and F at the third position, was shown to restore growth and subcellular trafficking by preventing *mur3* intracellular aggregations, possibly by improving XyG solubility (Hoffmann & McFarlane, 2024). In this context, the aim of the present study was to investigate further to what extent XyG side chain complexity could impact cell wall mechanical properties and seed germination and explore the link with free XGO release and XyG trafficking. For this purpose, we analyzed dormancy phenotypes of double and triple mutants combining *axy8*, *bgal10*, and *xyl1* mutations, together with XyG biosynthesis mutants exhibiting differences in side chain length. The comparative analysis of these genotypes reveals that dormancy depth is not correlated with differences in complexity of side chains. We show that the very specific effect of *xyl1* on seed dormancy in single and multiple mutants is clearly associated with accumulation of free oxidized XGO and altered XyG localization in cell walls. These results suggest a strong link between XGO recycling, XyG trafficking and dormancy maintenance.

## RESULTS

### Xylose removal has a prominent function in seed dormancy

**Figure 1:**
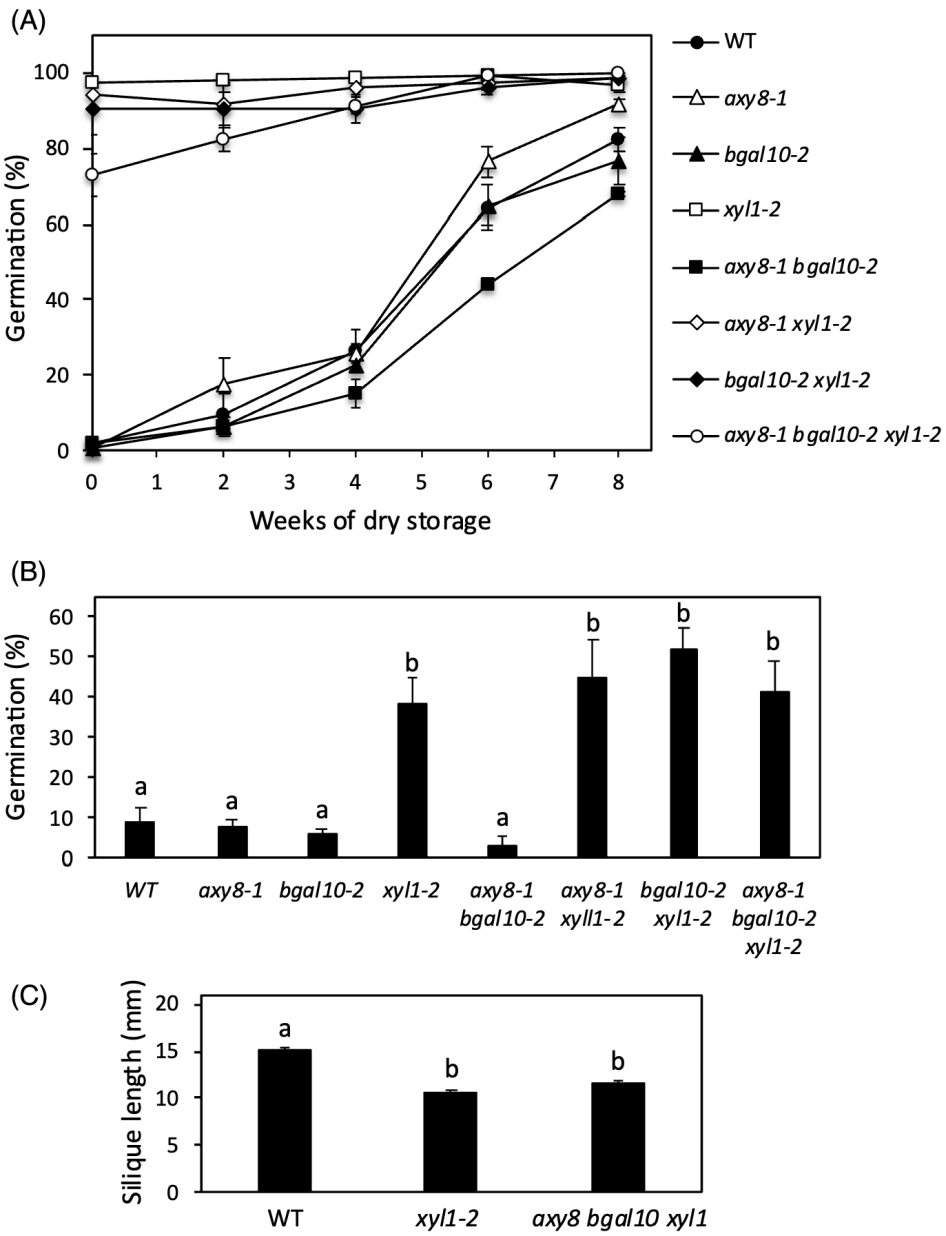
Seed dormancy, paclobutrazol resistance, and silique length in wild type and *axy8*, *bgal10*, *xyl1* single and multiple mutants. (A) Germination (radicle protrusion) of dry mature seeds stored during 0 to 8 weeks at room temperature was scored 2 weeks after sowing. Means of three biological replicates are shown with SE. (B) Germination (green cotyledons) of stratified seeds was scored 1 week after sowing in the presence of 30 μM paclobutrazol. Means of three technical replicates are shown with SE. Two independent experiments were performed with similar results. (C) Reduction of silique length in *xyl1‐2* and *axy8‐1 bgal10‐2 xyl1‐2* mutants compared to wild type (WT). Means of 8 biological replicates are shown with SE. (B, C) Statistical analysis was performed using one‐way ANOVA analysis with Tukey's HSD test (*P* < 0.05). Different letters indicate statistically significant differences.

Freshly harvested seeds of wild‐type (Col‐0) Arabidopsis seeds are dormant and do not germinate at 25°C in the light. In a previous study (Sechet et al., 2016), we observed that loss‐of‐function of the α‐xylosidase XYL1 reduced seed dormancy whereas *bgal10* and *axy8* mutations did not. This has been confirmed here using two independent alleles of each mutant (Figures S2 and S3A). Dormancy can be released by a few weeks of after‐ripening at room temperature and the time of storage necessary for a complete dormancy release is a good indicator of dormancy depth (Soppe & Bentsink, 2020). Then, we analyzed the impact on dormancy of combinations of *axy8* and *bgal10* mutations with *xyl1*, by scoring, every 2 weeks during storage, germination of seed lots of single and multiple mutants harvested on plants grown at the same time in a greenhouse (Figure 1A). Seed germination rates of freshly harvested seeds of double and triple mutants containing the *xyl1‐2* mutation were high, close to 100%, as observed in the single mutant *xyl1‐*2, indicating the absence of dormancy in these mutants. In contrast, in *axy8‐1*, *bgal10‐2*, and *axy8‐1 bgal10‐2* mutants, dormancy was similar to wild type. The *xyl1* mutation has also been shown to induce an increased germination resistance to paclobutrazol that indicated a lower GA requirement for germination activation (Sechet et al., 2016). In all mutant combinations containing the *xyl1‐2* allele (Figure 1B), a correlation was observed between the reduced dormancy and paclobutrazol resistance. The paclobutrazol resistance was similar in double and triple mutant seeds as compared to *xyl1*. Lack of dormancy and paclobutrazol resistance can be related to ABA deficiency or insensitivity. Previous studies showed that ABA content was slightly increased in *xyl1* dry seeds and slightly reduced in imbibed seeds at high temperature (Sechet et al., 2016; Shigeyama et al., 2016). Furthermore, we observed here that ABA sensitivity was not affected in *xyl1* germinating seeds (Figure S3B). Since dormancy and paclobutrazol resistance phenotypes in *xyl1* seeds were similar to those of severe ABA‐deficient or insensitive mutants, they likely could not result from observed hormonal defects. Reduction of silique length is another reproductive phenotype that has been reported in *xyl1* (Sampedro et al., 2010) and the triple mutant exhibited a similar phenotype to *xyl1* (Figure 1C). Altogether, these results suggested that combinations of *axy8* or *bgal10* mutations with *xyl1* had neither additive nor reducing effects on the seed and silique phenotypes observed in *xyl1*.

### The abundance of highly branched XyG is not correlated with dormancy depth

In *xyl1* seeds, an increase in xylosylated and galactosylated residues at the expense of fucosylated residues has been previously observed (Sechet et al., 2016), as also reported in *xyl1* mutant stems and seedlings (Günl & Pauly, 2011; Sampedro et al., 2010). It has been hypothesized that the abundance of highly substituted residues in seed tissues may influence XyG interactions with cellulose microfibrils and/or modulate endosperm weakening by hydrolases (XTH, glucanases, endo‐β‐mannanases) or extensibility by expansins (Sechet et al., 2016). To correlate dormancy phenotypes and XyG composition in mutants defective in side chain trimming, relative subunit abundance has been analyzed by MALDI‐TOF mass spectrometry in both embryo and TE fractions. After 3‐h imbibition in water, seeds of each genotype were dissected and separated into embryo and TE before digestion by endoglucanase.

**Figure 2:**
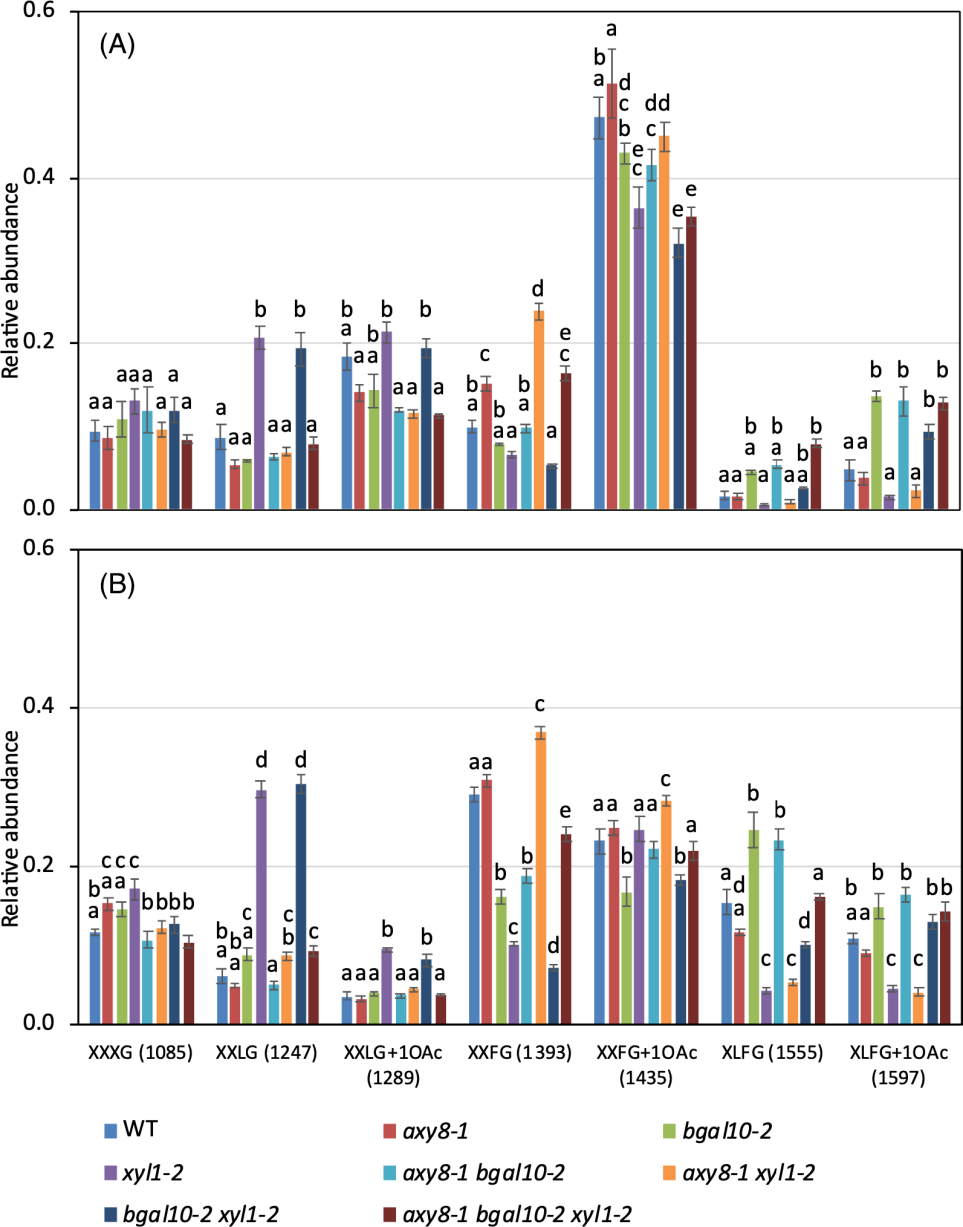
XyG relative abundance in wild type and *axy8*, *bgal10*, *xyl1* single and multiple mutant seeds. MALDI‐TOF analysis was performed on dissected seeds after imbibition during 3 h. (A, B) Embryos (A) were separated from testa and endosperm (B). Relative abundance of major subunits is shown as means of up to 6 biological replicates with SE. *m*/*z* are shown in brackets. Different letters indicate statistically significant differences as determined by two‐way ANOVA, followed by the post hoc Tukey's test (*P* < 0.05). Representative mass spectra are shown in Figure S4.

In all genotypes, XXFG (1393 & 1435 peaks) was the most abundant oligosaccharide in embryo fractions, representing about half of the total XyG. In TE, XXFG subunits were also abundant, but slightly less than in embryo (Figure 2). Furthermore, embryos differed from covering layers by the higher abundance in acetylated XXFG subunits, which represented about 40% of the total XyG content in wild‐type embryos and only 20% in TE. Another phenotype, which is common to all genotypes, was the higher relative abundance of highly substituted subunits XLFG in TE compared to embryo tissues (Figure 2; Figure S4).

Among single mutants, the XyG composition in *axy8‐1* did not show notable differences in major subunits compared to wild type, whereas in *bgal10‐2*, an increased XLFG abundance was observed at the expense of XXFG in both embryo and TE. As compared to *axy8* or *bgal10*, *xyl1* mutation induced stronger alterations in XyG composition. A reduction in XLFG subunits was particularly obvious in TE fraction, which was associated with an increased XXLG abundance. When combined to *axy8‐1* and to a smaller extent to *bgal10‐2*, *xyl1‐2* also reduced XLFG abundance in double mutants, as compared to the respective single mutants. Furthermore, when *bgal10‐2* was combined with *axy8‐1* or *xyl1‐2*, the increased abundance of XLFG in *bgal10‐2* was also observed in the double mutants compared to wild type and respective single mutants *axy8* and *xyl1*. The major effect of the combination of *axy8‐1* with *xyl1‐2* was to strongly increase the relative abundance of XXFG at the expense of XXLG, as compared to *xyl1‐2*. Interestingly, despite the diverse effects of single and double mutations on subunit abundance, the combination of the three mutations restored a XyG composition very similar to wild type in both embryo and TE fractions (Figure 2; Figure S4). Thus, the variations in XyG composition could not simply explain the differences in seed dormancy of the different mutant genotypes and the prominent effect of *xyl1*.

### Mutations in XyG biosynthesis differentially affect seed dormancy

**Figure 3:**
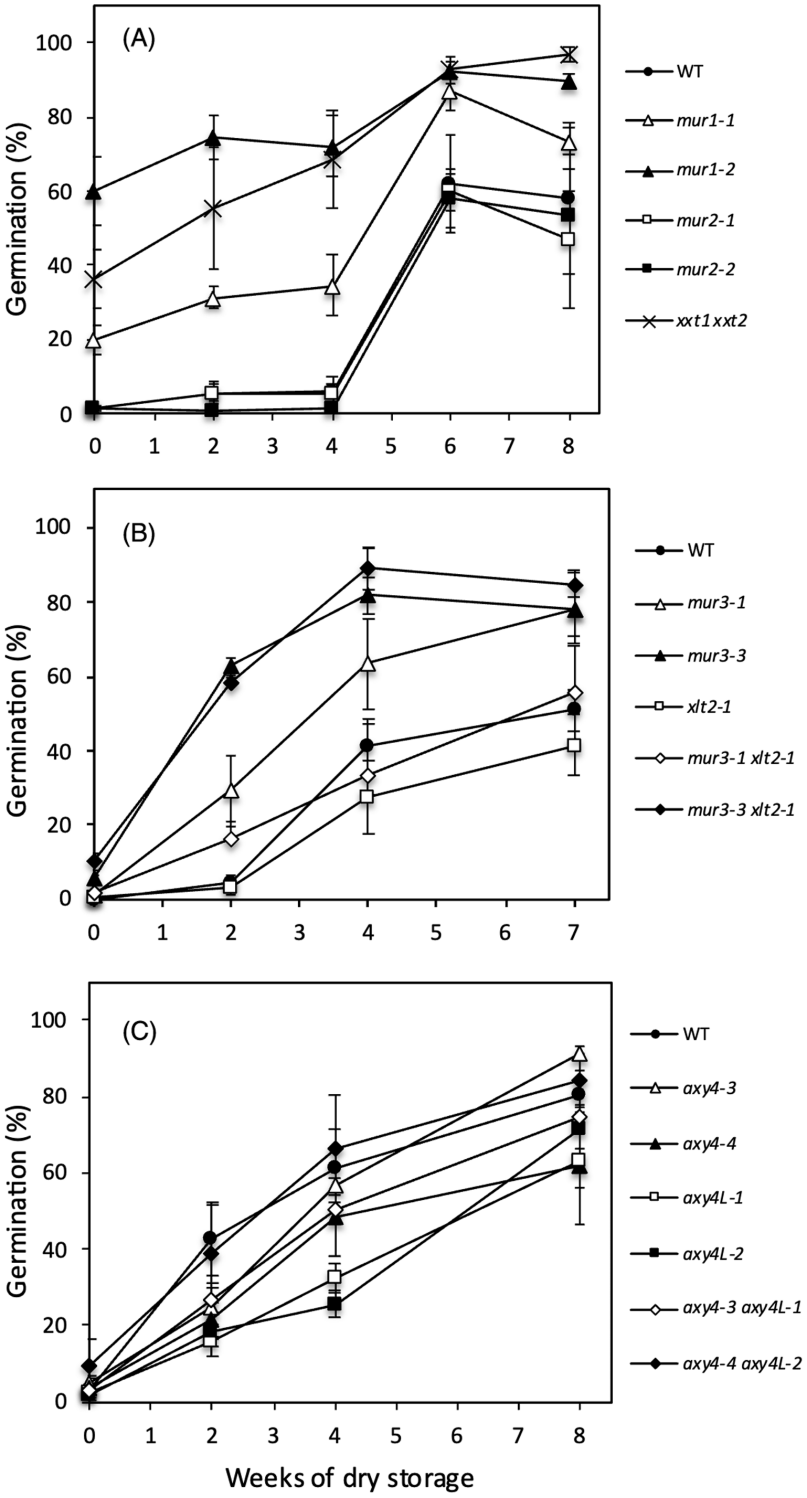
Germination of wild‐type and biosynthesis mutant seeds stored during 0 to 8 weeks at room temperature. (A) Fucosylation‐defective mutants *mur1* and *mur2* and xylosylation‐defective double mutant *xxt1 xxt2*, (B) Galactosylation‐defective mutants *mur3* and *xlt2*, (C) Acetylation‐defective mutants *axy4* and *axy4L*. Means of three biological replicates are shown with SE. Germination was scored 2 weeks after sowing. Two independent experiments were performed with similar results.

**Figure 4:**
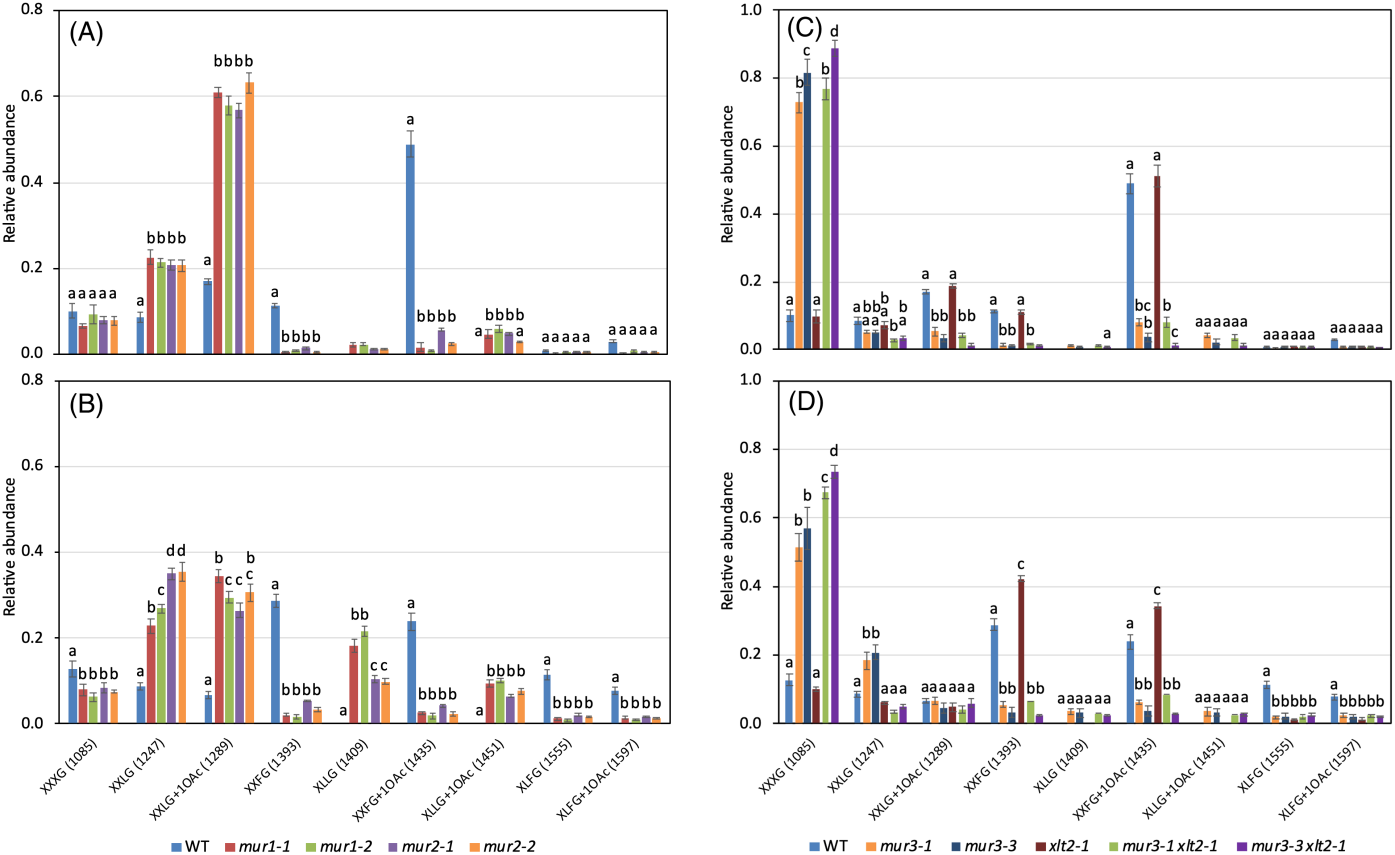
XyG relative abundance in wild‐type and biosynthesis mutant seeds. MALDI‐TOF analysis was performed on dissected seeds after imbibition during 3 h. (A–D) Embryos (A, C) were separated from testa and endosperm (B, D). Relative abundance of major subunits is shown as means of up to 6 biological replicates with SE. *m*/*z* are shown in brackets. Different letters indicate statistically significant differences as determined by two‐way ANOVA, followed by the post hoc Tukey's test (*P* < 0.05). Representative mass spectra are shown in Figures S4, S5, and S6.

To investigate further the relationship between seed dormancy and alterations in XyG composition, we investigated the impact of mutations in XyG biosynthesis affecting fucosylation, acetylation, galactosylation, and xylosylation (Figures S1 and S2). Freshly harvested seeds of the double *xxt1 xxt2* mutant, which lacks detectable XyG (Cavalier et al., 2008), exhibited a low level of dormancy since about 40% of them were able to germinate (Figure 3A). Thus, the production of XyG and its incorporation into cell walls would be required for seed dormancy establishment and/or maintenance. Together with the *xxt*, *mur1* seeds were less dormant upon harvest. In contrast, *mur2* seeds exhibited a dormancy similar to wild type (Figure 3A). Both *mur1* and *mur2* mutants, which are affected in XyG fucosylation, exhibited a similar XyG composition in dry seeds. Both embryo and TE tissues contained a higher amount of galactosylated residues (XXLG/XLXG and XLLG) at the expense of XXFG and XLFG, whose levels are close to background (Figure 4A,B; Figure S5). The *mur1* mutation has been shown to have pleiotropic effects on both XyG and pectin composition. Thus, as reported for vegetative phenotypes (Bonin et al., 2003; Bonin & Reiter, 2000), the reduced dormancy in *mur1* alleles compared to *mur2* could certainly be attributed to pectin alterations in seed tissues, suggesting no detectable impact of the absence of XyG fucosylation on seed germination.

As mentioned in the introduction, the *mur3‐3* allele impaired in XyG galactosylation has been reported to exhibit very strong vegetative defects. Indeed, this mutant developed as very dwarf and bushy plants and produced small siliques containing a very reduced number of seeds. In contrast, the leaky *mur3‐1* allele grew normally. Both alleles were crossed to *xlt2‐1* to obtain double mutants affected in galactosylation of both the third and second xylosyl residue of XXXG subunits (Figure 4C,D; Figure S6). Freshly harvested *mur3* and *mur3 xlt2‐1* seeds did not germinate, as observed for wild‐type seeds (Figure 3B). Nevertheless, after dry storage, dormancy release was faster in *mur3* seeds indicating a lower dormancy depth than in wild type. In accordance with the relative severity of the mutations, this phenotype was stronger in *mur3‐3* than in *mur3‐1*, which is close to wild type. Furthermore, the phenotype of the double mutant *mur3‐3 xlt2‐1* was similar to that of the single *mur3‐3* mutants, suggesting that the galactosylation by XLT2 of the second xylosyl residue did not impact on seed dormancy.

**Figure 5:**
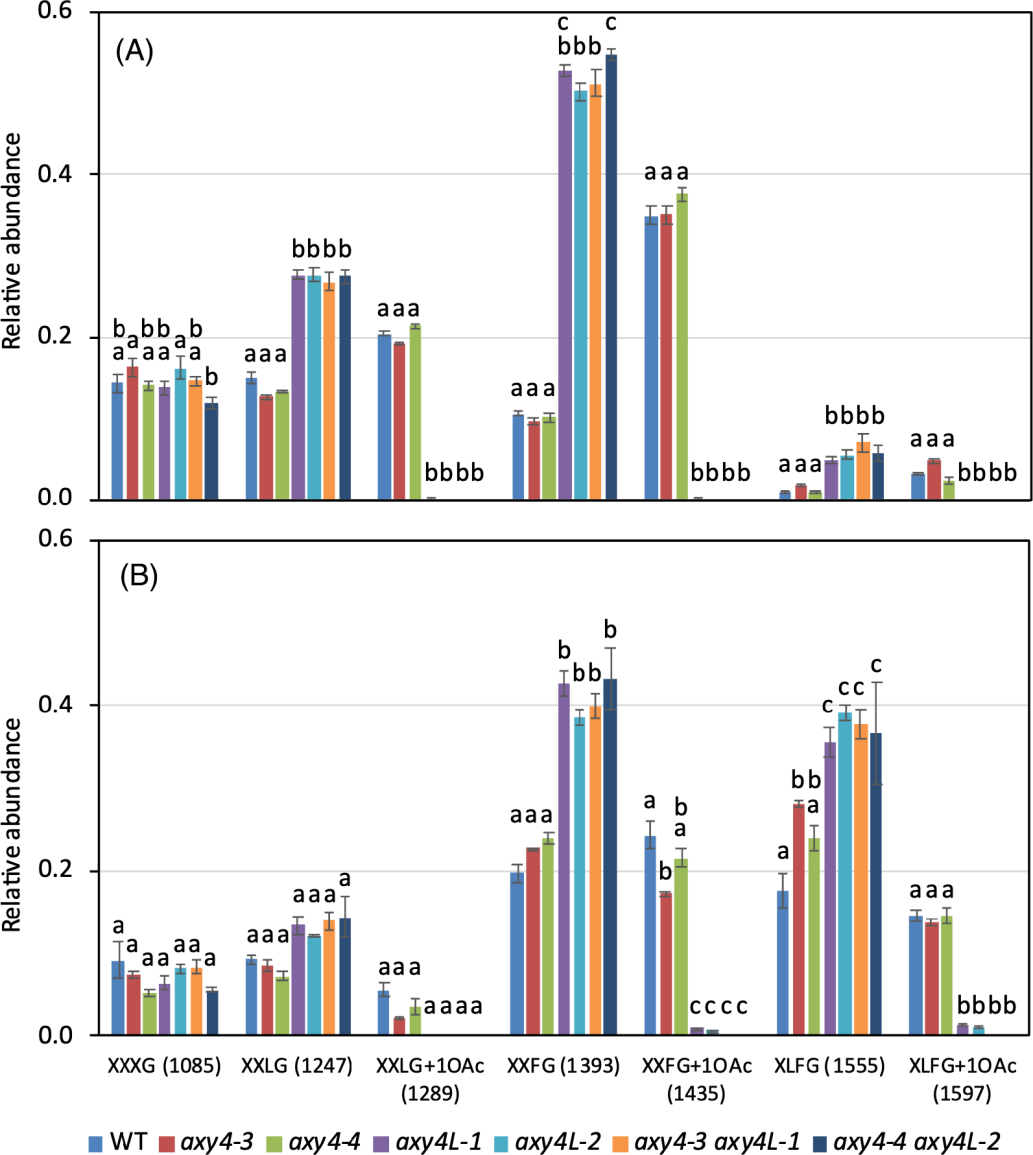
XyG relative abundance in wild type and acetylation‐defective *axy4* and *axy4L* single and double mutant seeds. MALDI‐TOF analysis was performed on dissected seeds after imbibition during 3 h. (A, B) Embryos (A) were separated from testa and endosperm (B). Relative abundance of major subunits is shown as means of up to 6 biological replicates with SE. *m*/*z* are shown in brackets. Different letters indicate statistically significant differences as determined by two‐way ANOVA, followed by the post hoc Tukey's test (*P* < 0.05).

The function of the *O*‐acetylation of XyG is not well understood and no effect of the absence of *O*‐acetyl‐substituents has been observed on vegetative development. Among the two *O*‐acetyl transferases that act on XyG, only AXYL would be active in seeds, since no acetylation of galactosyl residues has been detected in *axy4L‐1* mutant seeds (Gille et al., 2011). We confirmed here the specific absence of *O*‐acetylation in embryos of two independent *axy4L* alleles, however a residual accumulation of acetylated subunits was detected in TE tissues (Figure 5). Thus, we crossed two independent *axy4* alleles with *axy4L‐1* and *axy4L‐2* and a complete absence of acetylated subunits was observed in double mutants (Figure 5; Figure S2). All single and double mutants exhibited a dormancy similar to wild type (Figure 3C), therefore the physiological role of *O*‐acetylation in seeds remains elusive.

### XyG biosynthesis and remodeling genes have different expression patterns in seeds

**Figure 6:**
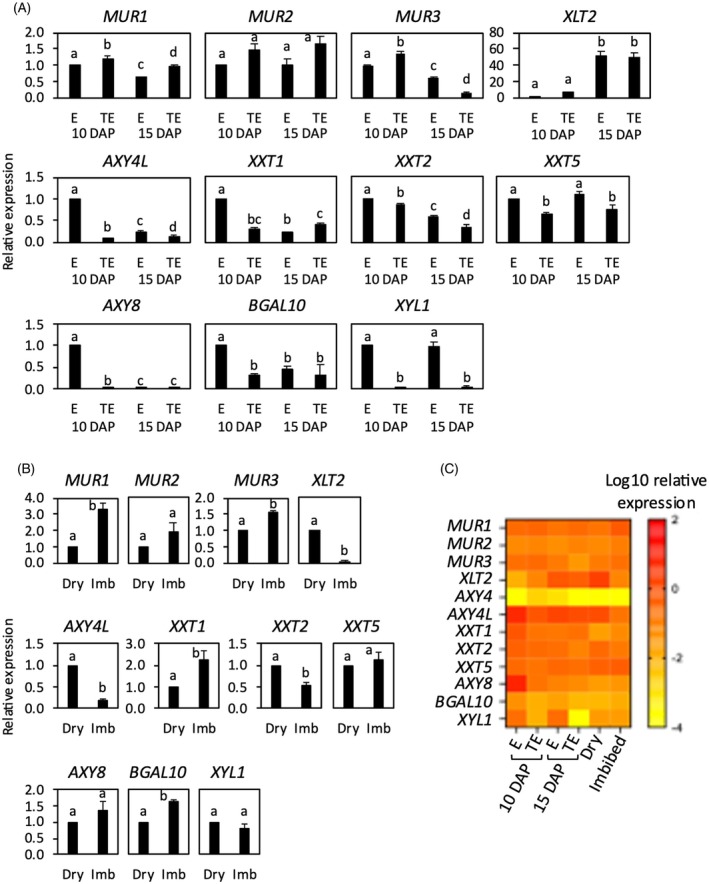
Relative expression levels of XyG metabolism genes in wild‐type seeds. (A) In embryo (E) and testa/endosperm (TE) fractions dissected from developing seeds at 10 and 15 days after pollination (DAP). (B) In dry and 3 h‐imbibed seeds. Means of three biological replicates are shown with SE. Expression levels were normalized with that of At4g12590 reference gene and with expression level in embryo at 10 DAP (A) or dry seed (B). (C) Heatmap of relative expression levels normalized with that of At4g12590 reference gene. For each gene, different letters indicate statistically significant differences as determined by (A) two‐way ANOVA, followed by the post hoc Tukey's test (*P* < 0.05) or (B) *t*‐test (*P* < 0.05). AXY4 expression levels were either undetected or too low to be included in A and B.

Differences in XyG composition between embryo and TE have been observed in wild‐type and mutant genotypes, such as the decreased relative abundance of XLFG subunits in embryo compared to TE. In wild‐type developing seeds, the expression of XyG metabolism genes has been measured in both tissue fractions (Figure 6) in order to establish correlations with XyG composition (Figures 2, 4, and 5). Quantitative PCR analysis indicated a higher accumulation of *AXY8* transcripts at 10 DAP in embryo compared to TE, in accordance with available expression data in the BAR database (Figure 6; Figure S7; https://bar.utoronto.ca/). Thus, a more active removal of fucosyl residues in the embryo could explain the higher relative abundance of XXLG at the expense of XLFG in wild‐type embryo fraction compared to TE (Figure 2). To a lesser extent, the higher expression of the other two glucosidases might also contribute to the lower abundance of highly branched XLFG subunits in the embryo. Another characteristic of XyG composition in embryo compared to TE was the higher abundance of acetylated residues, which could be correlated with the high expression of *AXY4L* in embryo tissues at 10 DAP (Figure 6; Figure S7). In contrast, *AXY4* transcripts were hardly detectable in both fractions (Figure 6C). The expression of other biosynthesis genes was relatively similar between embryo and TE and stable during seed development. Interestingly, *XLT2* transcript accumulation increased during late development and was higher than *MUR3* in both embryo and TE (Figure 6A,C). In good correlation, *XLT2* expression was also high in mature dry seeds (Figure 6C), suggesting a subtle regulation of XyG galactosylation during seed development. Upon 3‐h imbibition in water, major differences in transcript accumulation in imbibed seeds, compared to dry seeds, were observed for *XLT2* and *AXY4L* which strongly decreased (Figure 6B,C). These expression patterns may further suggest the importance of specific modifications of XyG structure during seed development, which otherwise were not observed to have any impact on seed germination.

### Seed dormancy phenotype is correlated with a defect in XyG localization

Together with seed dormancy and XyG composition, *xyl1* mutation has been shown to affect XyG accumulation in hypocotyl cell walls at endosperm rupture upon radicle emergence. During seed development and early imbibition, the immunolocalization of XyG epitopes by different specific antibodies revealed a strong and ubiquitous fluorescence signal in both longitudinal and transversal cell walls of wild‐type and *xyl1* seed tissues (Sechet et al., 2016). At endosperm rupture, an anisotropic accumulation was observed in wild‐type hypocotyls, XyG epitope abundance being low in longitudinal cell walls and high in transversal cell walls. In contrast, in *xyl1*, a low epitope abundance was detected in both transversal and longitudinal cell walls (Sechet et al., 2016). A similar immunolocalization analysis was performed here at endosperm rupture in imbibed seed sections, using the CCRCM1 antibody that binds XXFG. It confirmed the presence of an anisotropic distribution in wild‐type hypocotyl cell walls and its absence in *xyl1‐2* (Figure S8A,D).

**Figure 7:**
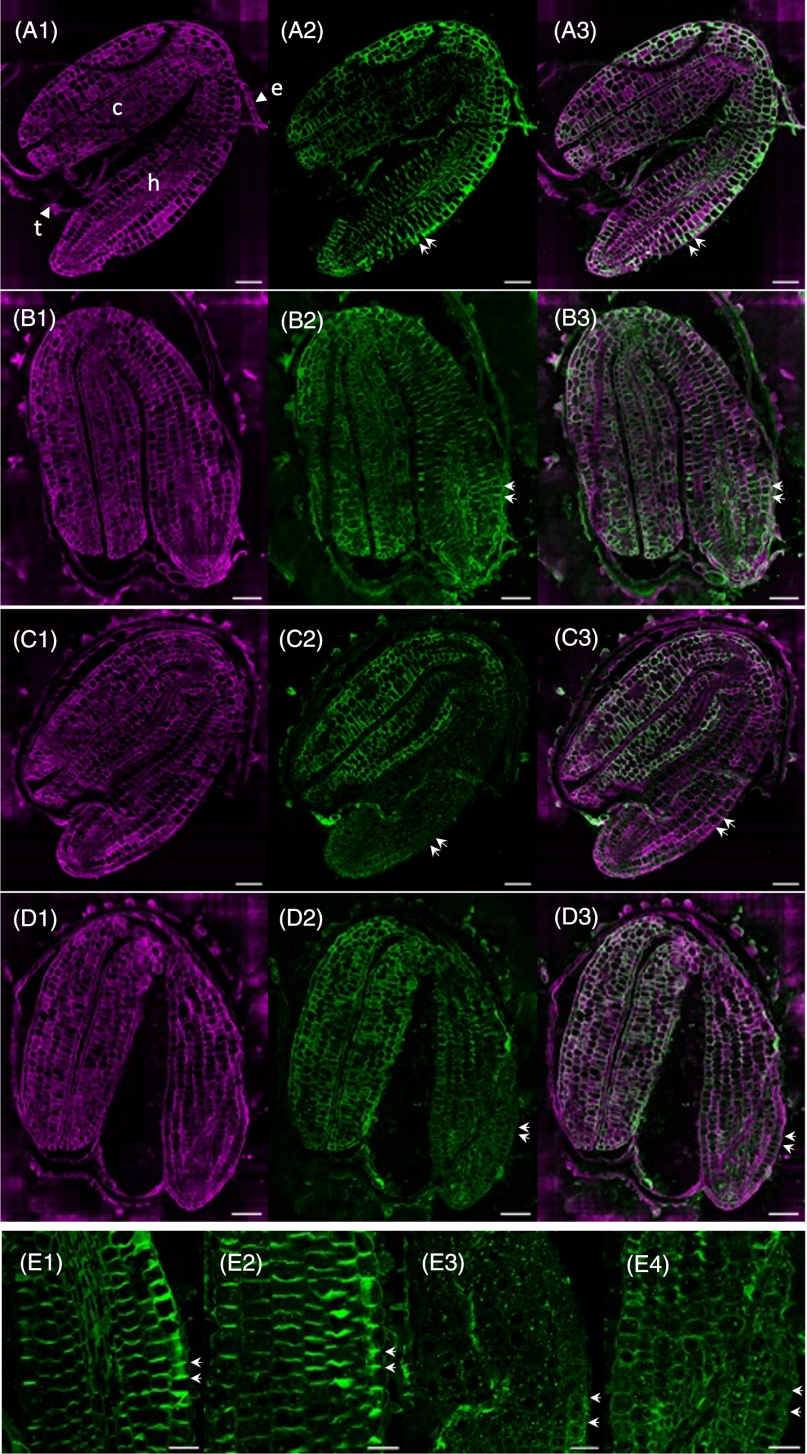
XyG immunolocalization in wild‐type and mutant germinating seeds at endosperm rupture. (A–D) Whole seed sections of wild type (A), *axy8‐1 bgal10‐2* (B), *xyl1‐2* (C), and *axy8‐1 bgal10‐2 xyl1‐2* (D). In magenta: cellulose labelling with calcofluor (A1–D1), in green: antibody labelling with CCRCM1 (A2–D2) and composite images showing both calcofluor and CCRCM1 labelling (A3–D3). Bar = 50 μm. Enlarged images of CCRCM1 labelling in wild‐type (E1), *axy8‐1 bgal10‐2* (E2), *xyl1‐2* (E3), and *axy8‐1 bgal10‐2 xyl1‐2* (E4) hypocotyls. Bar = 25 μm. c, cotyledons; e, endosperm; h, hypocotyl; t, testa. Double arrows highlight immunolabeling of transversal cell walls in embryo hypocotyl.

To determine whether this alteration of XyG localization was specific to *xyl1*, immunolabeling was performed on *axy8‐1* and *bgal10‐2* seed sections. In both mutants, labelling was similar to wild type (Figure S8A–C) and no difference was observed when labelling was performed with either CCRCM1 or LM25, the latter being specific of galactosylated (XXLG) residues (Figure S8E). To investigate further the correlation between dormancy phenotypes and the anisotropic distribution of XyG at germination, we compared the triple mutant *axy8 bgal10 xyl1* to the double mutant *axy8 bgal10* (Figure 7). The double mutant shows no defect in XyG localization in embryo hypocotyls, in contrast to the triple mutant which exhibits a low XyG abundance in both longitudinal and transversal cell walls, as observed in *xyl1*. Thus, the cleavage of xylose by XYL1 is essential to induce both an anisotropic distribution of XyG in cell walls and seed dormancy. Again, no correlation could be made between epitope anisotropy in cell walls and the complexity of XyG side chains.

### Altered XyG localization may be linked to free oligosaccharide release

**Figure 8:**
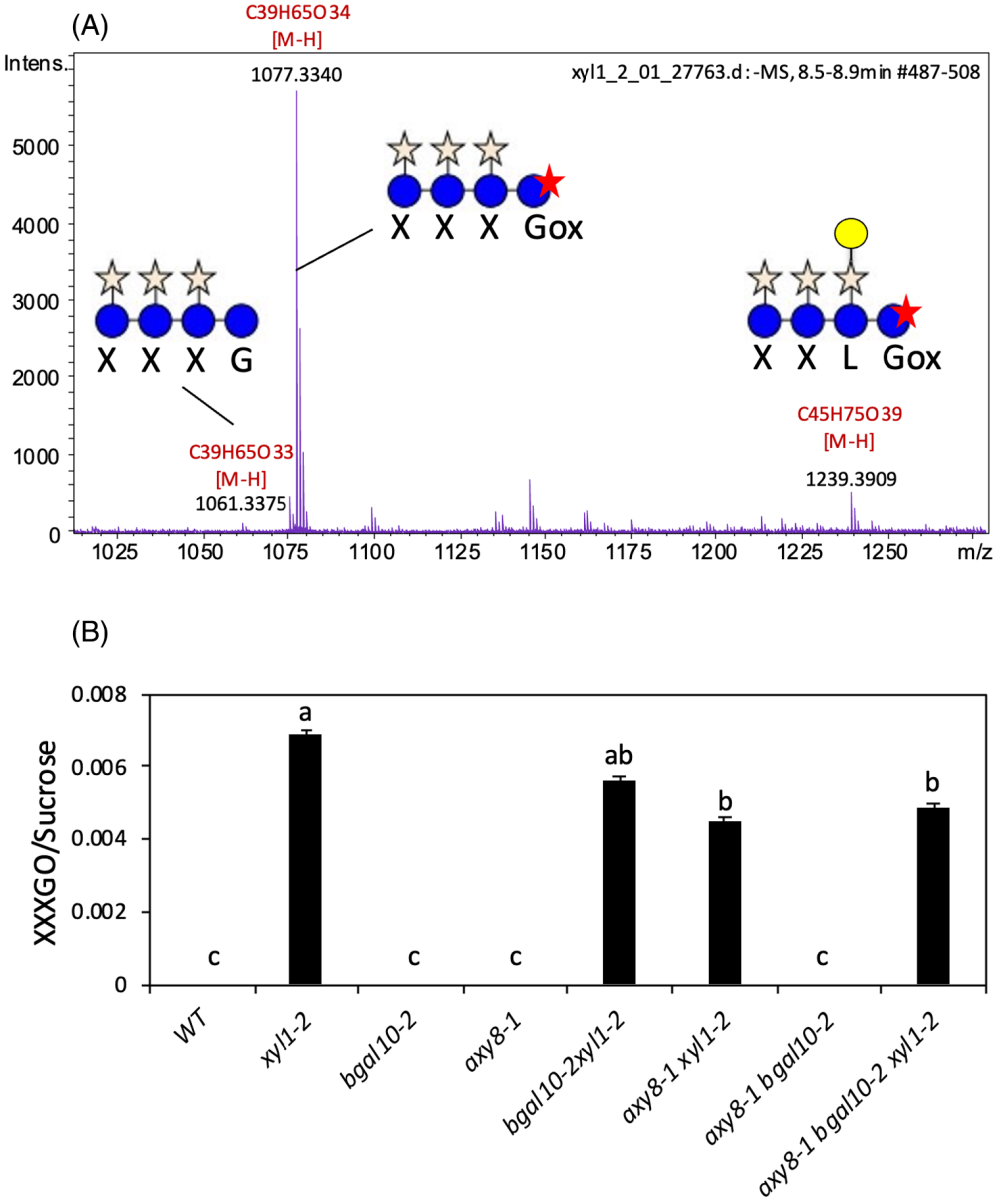
Accumulation of XXXG oligosaccharides in wild‐type and mutant seeds. (A) Spectrum of xyloglucan oligosaccharide‐enriched extract acquired in the negative ion mode, prepared from germinating seeds. Gox stands for oxidized glucose. (B) XXXGox peak area normalized to sucrose peak area. Data are means of three biological replicates with SE. Statistical analysis was performed using one‐way ANOVA analysis with Tukey's HSD test (*P* < 0.05). Different letters indicate statistically significant differences.

Free XGO (XXXG and XXLG) have been detected in the growth medium of *xyl1* mutants and in various tissues (stem, pericarp, seed, silique) (Di Marzo et al., 2022; Sampedro et al., 2010; Shigeyama et al., 2016). The impact of XGO accumulation on cell wall remodeling remains elusive. To determine whether mutations in other glycosidases AXY8 and BGAL10 affected free XGO accumulation, seeds of single and multiple mutants were stratified in water for 48 h at 4°C in the dark then transferred to 22°C during 36 h in the light. Extracts of germinating seeds were analyzed using high‐performance size‐exclusion chromatography (HP‐SEC) in conjunction with high‐resolution mass spectrometry (HRMS) in negative mode (Figure 8; Figure S9). We searched in *xyl1* samples for ions at *m*/*z* 1061, 1223, 1385, and 1531 [M−H]^−^, corresponding to the software‐predicted formula C₃₉H₆₅O₃₃ (indicative of XXXG), C_45_H_75_O_38_ (XXLG), C_51_H_85_O_43_ (XLLG), and C_57_H_95_O_47_ (XFLG), respectively. In *xyl1*, an ion at *m*/*z* 1061 was detected at a very low level alongside more intense ions at *m*/*z* 1077 and 1239, which matched the predicted formulae C₃₉H₆₅O₃₄ and C_45_H_75_O_39_, respectively (Figure 8A). The fragmentation of *m*/*z* 1077 ion resulted in the formation of ions at *m*/*z* 149 (pentosyl unit), 131 (anhydro‐pentosyl unit), and 293 [pentosyl‐hexosyl‐H]^−^ fragments that correspond to entire inner xyloglucan side chains (Quéméner et al., 2015). The ion at *m*/*z* 941, produced via a 136‐Da loss, was attributed to a ^2,4^A_4_ cross‐ring cleavage in a C4‐substituted hexose in an oxidized form. Indeed, a 120‐Da loss is usually observed upon ^2,4^A_4_ cross‐ring cleavage in a C4‐substituted non‐oxidized hexose (Lelas et al., 2024). Ions at *m*/*z* 647 and 353 were assigned to a ^2,4^A_3_ and ^2,4^A_2_ cross‐ring cleavage, respectively. Last, ions at *m*/*z* 545 and 233 were attributed to ^0,2^A_2_ and ^0,2^A_1_, respectively. This MS^2^ pattern, characteristic of xyloglucan oligosaccharide fragmentations, was therefore attributed to the oxidized form of XXXG (XXXGox). Although we were unable to obtain an exploitable fragmentation pattern for the ion at *m*/z 1239 and to detect it in all replicates due to its very low intensity, it likely corresponds to the oxidized form of XXLG (XXLGox). XXXGox was also found in *bgal10 xyl1*, *axy8 xyl1*, and *axy8 bgal10 xyl1* (Figure 8B) and, as in *xyl1*, more substituted free XGO were not detected.

## DISCUSSION

In contrast to many studies that describe the role of XyG in vegetative tissues, the impact of mutations in XyG metabolism on seed physiological processes has not been studied in great details. A few studies focused on XYL1 function in seeds (Di Marzo et al., 2022; Sechet et al., 2016; Shigeyama et al., 2016), however the complete absence of the three major apoplastic glycosidases had not been explored.

### Presence and abundance of xylose residues impact on seed germination

Among the XyG metabolism mutants tested, only five of them, *xxt1 xxt2*, *mur1*, *mur3*, *mur3 xlt2*, and *xyl1* exhibited a reduced dormancy and no mutations increased dormancy depth (Figures 1 and 3). Phenotypic comparison between *mur1* and *mur2* alleles suggested that *mur1* phenotype likely results from an altered pectin composition. Low seed dormancy of *mur1* seeds strongly supports the importance of pectin composition on germination processes, as reported for pectin methyl esterification (Müller et al., 2013; Scheler et al., 2015). All other four genotypes have an altered xylose content. The double mutant *xxt1 xxt2* is defective for the addition of xylose residues on the glucan backbone. XXT1 and XXT2 are responsible for the addition of the first two xylose residues and their simultaneous defect causes a complete lack of XyG (Cavalier et al., 2008). The absence of XyG has been shown to affect cell wall biomechanical properties, including cell wall loosening by expansins (Park & Cosgrove, 2012). Expansins have been shown to have an essential role in GA‐activated germination processes, such as in endosperm expansion prior to radicle protrusion (Sánchez‐Montesino et al., 2019). The lack of dormancy of *xxt1 xxt2* further supports the importance of XyG in mechanical resistance of seed tissues, which influence dormancy induction or maintenance. Possible compensatory mechanisms by other polysaccharides have been suggested to explain the mild plant growth defects in *xxt1 xxt2* (Bou Daher et al., 2024; Sowinski et al., 2022), however these would not be sufficient to reinforce endosperm resistance and/or prevent embryo growth.

In contrast to *xxt1 xxt2*, freshly harvested seeds of *mur3* and *mur3 xlt2* mutants were not able not germinate and their reduced dormancy could only be detected upon after‐ripening. Thus, the increased abundance in XXXG subunits in *mur3* mutants could be correlated with lower dormancy. The simultaneous loss‐of‐function of XLT2 did not further reduce seed dormancy, despite a slightly higher abundance in XXXG subunits in *mur3‐3 xlt2‐1* compared to *mur3‐3* (Figure 4). Nevertheless, it is surprising that despite the severe growth defects reported in *mur3* mutants (Kong et al., 2015), seed germination processes were only mildly affected.

The strongest reduction in seed dormancy was observed in *xyl1* which exhibits a minor increase in the relative abundance of XXXG subunits compared to *mur3* (Figures 2 and 4). Thus, more than variations in XXXG abundance, it appears that the removal of xylose residues is essential for cell wall remodeling in seeds. Several isoforms of XYL1 protein have been shown to accumulate in dry seeds and overexpression of XYL1 under the control of an endosperm‐specific promoter to restore dormancy and even delay radicle protrusion in *xyl1* transgenic seeds (Sechet et al., 2016). Altogether, these results indicate that XyG have an essential role in cell wall remodeling in seeds and xylose removal could be a key regulatory step to maintain dormancy and fine‐tune germination.

### XyG side chain length does not influence dormancy phenotypes

The absence of seed dormancy in *xyl1* mutants has been suggested to be linked to the low abundance of the highly branched XyG, which might affect XyG interactions with cellulose microfibrils (Sechet et al., 2016). In accordance, dormancy depth in *axy8* and *bgal10* seeds is similar to wild type and relative abundance of XLFG subunits is equal or higher than wild type (Figures 1 and 2). In contrast, dormancy depth in triple mutant *axy8 bgal10 xyl1* seeds is as low as in *xyl1*, but XyG composition is close to that of wild type. Furthermore, the strong reduction in fucosylated residues observed in both *mur2* and *mur3* has little or no effect on seed dormancy (Figures 3 and 4). Despite some studies that reported an enhanced binding of fucosylated XyG to crystalline cellulose surface, as reviewed by Park and Cosgrove (2015), our results do not show any clear correlation between XyG ramification complexity and seed germination phenotypes.

Apoplastic glycosidase activities have been suggested to be responsible for structural diversity of XyG and their absence to limit XyG turnover, as reported in the *axy8 xyl1* double mutant (Günl et al., 2011). Thus, it is somewhat surprising that the loss‐of‐function of the three major glycosidases has a very limited effect on the relative abundance of XyG subunits. In particular, a greater abundance of XLFG subunits would be expected in the triple mutant *axy8 bgal10 xyl1*. Since apoplastic modifications occur after XyG translocation from the Golgi, their abundance likely depends on the degree of substitutions before secretion into the wall and may also be limited by the steric hindrance in polysaccharidic chains. Besides, the existence of a functional redundancy of both BGAL10 and AXY8 has been suggested since mutant phenotypes were mild and residual galactosidase and fucosidase activities were detected in *bgal10* and *axy8* mutants, contrasting with the complete absence of xyloxidase activity in *xyl1* (Günl et al., 2011; Sampedro et al., 2010, 2012). Thus, residual XyG modifications in the absence of these glycosidases cannot be excluded and may also limit the abundance of highly substituted residues. In any case, if side chain length modifies XyG interactions with other components of the cell wall such as cellulose microfibrils, it does not have an obvious impact on mechanical properties of seed tissues involved in dormancy.

### XYL1 is involved in polarized XyG turnover

Defects in XyG biosynthesis or degradation have been shown to affect XyG intracellular localization. The *mur3‐3* mutant that is defective for XXXG galactosylation at the third residue has been shown to accumulate cell wall polysaccharides, including XyG, in intracellular aggregates, and to be impaired in the organization of endomembranes and actin filaments (Kong et al., 2015; Tamura et al., 2005). Growth defects and endomembrane aggregation were largely suppressed in *xxt2 mur3* and *xxt5 mur3*, suggesting that a high proportion of unsubstituted XXXG is dysfunctional and may cause XyG aggregation in the endomembrane system and perturb plant development (Kong et al., 2015). Furthermore, the replacement of galactose by d‐galacturonic acid onto XyG was shown to restore *mur3* vegetative growth and prevent XyG aggregation suggesting that side chain length was important (Hoffmann & McFarlane, 2024).

The *xyl1* mutant has only mild growth defects, compared to *mur3‐3*. Nevertheless, as compared to wild type, an accumulation of intracellular vesicles labeled with anti‐XyG antibodies has been observed in *xyl1* hypocotyls at endosperm rupture, together with the absence of XyG labelling in transversal cell walls (Sechet et al., 2016). As shown here (Figure 7; Figure S8), these defects in XyG intracellular localization were not observed in *axy8*, *bgal10*, and *axy8 bgal10*, suggesting a specific role of xylose removal in XyG trafficking. The combination of *xyl1* with *axy8 bgal10* also prevented the accumulation of XyG in transversal cell walls upon hypocotyl elongation, ruling out a positive role of side chain length. No difference in the perturbations in XyG localization has been observed in the triple mutant compared to *xyl1*, as well as in dormancy phenotypes, suggesting a minor role of AXY8 and BGAL10 and supporting a strong link between intracellular XyG mislocalization and germination phenotypes.

During seed development and early imbibition, a ubiquitous XyG accumulation in all cell walls has been observed in *xyl1* embryo and endosperm, as in wild‐type (Sechet et al., 2016). At endosperm rupture (Figure 7), in wild‐type seeds, increased XyG labelling in transversal cell walls and its absence in longitudinal walls can be attributed to specific hydrolysis and remobilization from longitudinal walls. XyG degradation in longitudinal cell walls may favor hypocotyl elongation. In *xyl1*, reduction of XyG labelling in longitudinal cell walls suggests that hydrolysis is active but remobilization, through endocytosis and secretion, to transversal cell walls could be impaired. No detrimental effect on embryo growth has been observed and germination is even facilitated, however the absence of polarized XyG localization in embryo cell walls can be correlated with reduced anisotropic growth, as observed in fruit and sepal (Günl & Pauly, 2011; Sampedro et al., 2010). Detection of labeled XyG in endosperm cell walls upon its rupture could not be achieved. Nevertheless, since XYL1 overexpression specifically in the endosperm suppressed germination defects (Sechet et al., 2016), it can be hypothesized that XyG turnover and cell wall elasticity could be altered in *xyl1* endosperm. Direct assessment of the impact of *xyl1* mutation on biomechanical properties of Arabidopsis seeds remains challenging, however other organs have been previously analyzed. Despite meristem size was affected, atomic force microscopy (AFM)–based nano‐indentation did not detect any differences in cell wall stiffness between *xyl1* and wild‐type meristems (Zhao et al., 2019). Nevertheless, AFM measurements on elongating siliques revealed an increased stiffness of valve cells (Di Marzo et al., 2022) and creep‐extension analysis a reduced viscoelasticity of elongating stems (Shigeyama et al., 2016). Puncture force method has been used to assess endosperm weakening of germinating seeds in several species, however it could not yet be used on Arabidopsis tiny seeds (Steinbrecher & Leubner‐Metzger, 2017).

### Free XGO oxidation may impair XyG turnover and cell wall remodeling

Accumulation of free XXXG in *xyl1* tissues has previously been reported, including in germinating seeds (Di Marzo et al., 2022; Sampedro et al., 2010; Shigeyama et al., 2016). Interestingly, we observed here that the concomitant absence of other glycosidases in double and triple mutants did not reduce XGO release (Figure 8). Free XGO other than XXXG were under detection limit, despite XyG cleavage by XTH would be expected to release more substituted subunits if degradation by any of the three major glycosidases cannot occur. This suggests that highly substituted subunits may be less easily released because of stronger binding to cellulose and/or XTH cleavage preferentially produces XXXG subunits. Alternatively, it can be hypothesized that highly substituted subunits may be more efficiently recycled or, in contrast to xylosidase, galactosidase, and fucosidase activities other than BGAL10 and AXY8 can act on free XGO.

Intriguingly, in germinating seeds, oxidized XXXG represented a large fraction of free XGO (Figure 8), unlike XGO released in the culture medium of 7‐day‐old Arabidopsis seedlings, which *m*/*z* (1085) corresponded to XXXG sodium adducts (Sampedro et al., 2010). MALDI‐TOF analysis of *xyl1* developing fruits detected free XXXG at a higher *m*/*z* (1101), which were considered as potassium adducts (Shigeyama et al., 2016). Here, fragmentation analysis clearly proved that 16 Da higher mass XXXG in germinating seed extracts were oxidized XGO (Figure S9). Thus, free XGO accumulated in cell walls of *xyl1* dry seeds, and possibly also in developing fruits, are subjected to oxidation events.

Oligosaccharides derived from plant cell walls upon pathogen attack or mechanical damage are named damage‐associated molecular patterns (DAMPs). DAMPs activate plant immunity and some of them also behave as negative regulators of growth and development. Control mechanisms to prevent deleterious physiological effects and hyper‐immunity caused by DAMP overaccumulation have been reported to involve specific oxidases belonging to the Arabidopsis berberine bridge enzyme‐like protein family. Arabidopsis members of this family have been shown to inactivate oligogalacturonides and cellodextrins released from pectin and cellulose respectively (Pontiggia et al., 2020). Moreover, mutations in two other members of this protein family have been reported to induce alterations in seed coat structure and mucilage release (Costantini et al., 2024). Oxidase activities that would act on XGO are unknown, nevertheless current knowledge supports that XGO would function as signaling molecules (Claverie et al., 2018; González‐Pérez et al., 2014), implying that inactivation mechanisms may exist to prevent excessive cellular response. Oxidation reactions of DAMPs can lead to the production of H_2_O_2_ (Pontiggia et al., 2020) and reactive oxygen species (ROS) have been widely reported to regulate seed germination, notably by interacting with hormone signaling or activating cell elongation and cell wall loosening (Bailly, 2019). Thus, it cannot be excluded that local ROS accumulation may contribute to dormancy release and/or germination activation.

The excess amount of XGO in *xyl1* has been suggested to interfere with endotransglycosylase activity and inhibit grafting of XyG chains, because XGOs might be incorporated into newly digested XyG instead of XyG chains, thus reducing their size and possibly affecting interactions with other cell wall components (Shigeyama et al., 2016). Indeed, XTH activities certainly have a prominent role in fine‐tuning germination. Loss of function of AtXTH31/XTR8, which expression is endosperm‐specific, has been reported to lead to faster germination, suggesting that AtXTH31/XTR8 is involved in the reinforcement of the cell wall of the endosperm during germination (Endo et al., 2012). XGO oxidation may reduce recycling by XTH and limit the available XXXG pool for XyG chain grafting. The excessive abundance of oxidized XGO could also favor persistence in intracellular vesicles and reduce XyG turnover and remobilization in both embryo and endosperm.

Altogether, this study highlights variations in XyG structure between embryo and protective layers, whose physiological significance remains elusive. Moreover, it is particularly intriguing that XyG complexity is not correlated with modifications of cell wall mechanical resistance that would affect seed germination. Apart from xylose removal, most modifications in XyG composition have a limited impact on seed dormancy. It is clearly shown here that xylosidase activity is essential for polysaccharide intracellular trafficking. Accumulation of free XXXG and inactivation by oxidation likely perturb XGO remobilization and polarized secretion in cell walls, which would negatively impact on anisotropic growth in elongating tissues and, in seeds, possibly modify hypocotyl growth and endosperm resistance. Further work would be necessary to decipher intracellular processes involved in XyG turnover and polarized accumulation and contribution of XGO oxidation reactions in cell wall remodeling during germination.

## MATERIAL AND METHODS

### Plant material and growth conditions

Arabidopsis wild‐type and mutant seeds (Columbia‐0 accession) were surface sterilized, sown in Petri dishes containing Arabidopsis Gamborg B5 medium (Duchefa, http://www.duchefa.com) supplemented with 30 mM sucrose, and stratified at 4°C in the dark for 3 days. Petri dishes were then placed for 4 days in a growth chamber (16‐h photoperiod, 50 μmol m^−2^ sec^−1^ light intensity, 18°C, 60% relative humidity). Germinated seedlings were transferred to soil (Tref Substrates, http://www.trefgroup.com) and unless otherwise stated, grown in a glasshouse with a minimum photoperiod of 13 h, assured by supplementary lighting. In each experiment, all genotypes were grown together. Mutants (Figure S2) were obtained from the GABI‐KAT (Kleinboelting et al., 2012), SALK (Alonso et al., 2003), and SAIL (Sessions et al., 2002). EMS mutants, *mur1‐1* and *mur1‐2*, were reported in Bonin et al. (1997), *mur3‐1* in Madson et al. (2003), *axy8‐1* in Günl et al. (2011), and *xyl1‐4* in Sechet et al. (2016).

### Germination experiments

For dormancy assays, freshly harvested seeds were stored at room temperature for 8 weeks. During storage, seeds were sown every 2 weeks in triplicate, in Petri dishes containing 0.5% (w/v) agarose, and then placed in a growth chamber (continuous light, 25°C, 70% relative humidity). Germination was scored 14 days after sowing based on radicle protrusion. For paclobutrazol resistance tests, seeds were sown on 0.5% (w/v) agarose supplemented with paclobutrazol (Syngenta, http://www.syngenta‐agro.fr), stratified at 4°C for 3 days in the dark, then incubated in the same conditions as the dormancy assays for 4 days. Seedlings were scored as resistant if they developed green cotyledons. All mother plants were grown together in a glasshouse and three independent seed lots harvested for each genotype, each seed lot was obtained by pooling seeds from 3 to 4 plants. Two independent cultures were performed.

### Xyloglucan analysis by MALDI‐TOF MS

Xyloglucan analysis was performed by enzymatic oligosaccharide fingerprinting, as previously described (Lerouxel et al., 2002; Sechet et al., 2016). Extracts were prepared from the six independent seed lots per genotype, which were used in the germination tests described above. After seed imbibition during 3 h in water, embryos were separated from testa and endosperm by dissection and placed in 96% ethanol. After ethanol removal and rehydration, xyloglucan oligosaccharides were generated by treating samples with desalted endoglucanase (Megazyme, E‐CELTR) in 0.01 M sodium acetate pH 5, overnight at 37°C. The samples and the super‐DHB matrix were deposited successively on MTP 384 ground steel BC targets (Bruker Daltonics, Bremen, Germany). Acquisition of xyloglucan oligosaccharide spectra was recorded with a MALDI/TOF reflex III mass spectrometer for *axy4* and *axy4L* mutants and a MALDI‐TOF/TOF UltrafleXtreme mass spectrometer (Bruker Daltonics) was used for all other experiments. Mass spectra were obtained in reflector‐positive ion mode. The laser intensity was set just above the ion generation threshold to obtain peaks with the highest possible signal‐to‐noise (*S*/*N*) ratio without significant peak broadening. All data were processed using the FlexAnalysis software package (Bruker Daltonics). Quantitative analysis of the data was performed using the packages MALDIquant (v1.22.1) and MALDIquantForeign (v0.14) in R (v4.3.1) (Gibb & Strimmer, 2012). Only major peaks corresponding to sodium adducts have been quantified, XXXG (1085), XXLG/XLXG (noted XXLG, 1247), XXFG (1393), XLLG (1409), XLFG (1555) and acetylated subunits, XXLG (1289), XXFG (1435), XLLG (1451), XLFG (1597) as indicated in Figures S4–S6.

### Free oligosaccharide analysis

Seeds (2 mg) from three independent seed lots per genotype were imbibed in Eppendorf tubes containing 50 μL sterile water. After stratification during 48 h at 4°C in the dark, tubes were incubated during 36 h at 22°C in the light. Seed pellets were grinded in 20% ethanol and final ethanol concentration adjusted to 70%, before centrifugation at 10 000*g* for 40 min. After supernatant evaporation, seed extracts were resuspended in 50 μL of deionized water and oligosaccharides separated according to Voxeur et al. (2019). Chromatographic separation was performed on an ACQUITY UPLC Protein BEH SEC Column (125 Å, 1.7 μm, 4.6522 mm × 300 mm, Waters Corporation, Milford, MA, USA) coupled with a guard Column BEH SEC Column (125 Å, 1.7 μm, 4.6 mm × 30 mm). Elution was performed in 50 mM ammonium formate, 0.1% formic acid at a flow rate of 0.4 mL min^−1^, with a column oven temperature of 40°C. Injection volume was set to 10 μL. Quantitative evaluation of cell wall fragments was conducted using an HPLC system (UltiMate 3000 RS HPLC system, Thermo Scientific, Waltham, MA, USA) coupled to an Impact II Ultra‐High Resolution Qq‐Time‐Of‐Flight (UHR‐QqTOF) spectrometer (Bruker Daltonics) equipped with an electrospray ionization (ESI) source in negative mode. The end plate offset set voltage was 500 V, capillary voltage was 4000 V, nebulizer pressure was 40 psi, dry gas flow was 8 L min^−1^, and the dry temperature was set to 180°C. The Compass 1.8 software (Bruker Daltonics) was used to acquire data, and peak areas were integrated manually. XXXG and XXXGox were annotated based on accurate mass annotation, isotopic pattern, and MS/MS analysis. Extracted ion chromatogram of XXXGox was integrated for the WT and various mutants.

### Expression analysis

Developing seeds were dissected from siliques, 10 and 15 days after tagging of flowers at fertilization stage, and separated into embryo and covering tissues composed of testa and endosperm. The samples were then immediately frozen in liquid nitrogen. Dry seeds and 3 h‐imbibed seeds were also frozen in liquid nitrogen. Total RNAs of 3 biological replicates were extracted by 20–50% isopropanol methods as described in Martin et al. (2005) with subsequent washing by Ethachinmate (NIPPON GENE, https://www.nippongene.com/26mmunol/index.html). Total RNA (1 μg) was used as a template to synthesize cDNA. Reverse transcription was performed using PrimeScript RT reagent kit with gDNA Eraser (Takara). Quantitative real‐time PCR reactions were performed using the Mx3000P QPCR System (Agilent Technologies) according to the previous study (Watanabe et al., 2020). Gene‐specific primers are listed in Table S1.

### Xyloglucan immunolocalization

Seeds were sown on filters and placed in a growth chamber (continuous light, 25°C, 70% relative humidity). About 20 h after transfer to 25°C, seeds that had undergone endosperm rupture were selected at the start of radicle protrusion. Samples were fixed in a solution of 4% (w/v) formaldehyde and 0.1% Triton X‐100 under vacuum for 1 h. Samples were then washed three times in phosphate‐buffered saline (PBS), dehydrated through a graded ethanol series (30, 50, 70, 90, and 97% (v/v) in PBS), and then incubated in a mixture of 100% wax and 97% ethanol (1:1, v/v) at 40°C overnight, and embedded in 100% wax at 40°C. Sections (8 μm) were prepared using a microtome and air‐dried onto polylysine‐coated glass slides. Samples on slides were dewaxed and rehydrated through a degraded ethanol series [97, 90, and 50% (v/v) in PBS]. Samples were blocked with 1% (w/v) bovine serum albumin in PBS (blocking solution) and incubated with LM25 or CCRC‐M1 in blocking solution. After three washes of 5 min each in PBS, slides were incubated with anti‐mouse IgG labeled with Alexa 488 (Molecular Probes, Eugene, OR, USA) in blocking solution. Following antibody labelling, the samples were washed in PBS and counterstained for 10 min with Calcofluor White M2R (fluorescent brightener 28; Sigma‐Aldrich) at 5 mg mL^−1^. After washing with PBS, slides were sealed. Immunofluorescence was observed using a spectral confocal laser‐scanning microscope (Zeiss LSM710). Image analysis was performed using ImageJ and an attenuation Plugin used to rectify Calcofluor White M2R fluorescence (Biot et al., 2008).

## AUTHOR CONTRIBUTIONS

AM‐P conceived the research. AM‐P, AV, GM, and MS supervised the experiments. HS, PS, and AF performed mutant genotyping and germination experiments. HS, PS, JS, and AV performed mass spectrometry experiments. LM‐P and AV analyzed data and performed statistical analysis. HS performed quantitative PCR analysis. AB, KB, and NB performed cytological and confocal microscopy experiments. AM‐P wrote the article.

## CONFLICT OF INTEREST

The authors declare no conflicts of interest.

## Supporting information


**Figure S1.** Nomenclature and schematic representation of XyG showing enzymes involved in their synthesis and modification, as detailed in the text.
**Figure S2.** Schematic diagram of XyG metabolism genes and mutants.
**Figure S3.** Dormancy and ABA germination inhibition in mutant seeds compared to wild‐type (WT).
**Figure S4.** MALDI‐TOF mass spectra of *axy8‐1 bgal1‐2 xyl1‐2* compared to *xyl1‐2* and wild type seeds.
**Figure S5.** MALDI‐TOF mass spectra of *mur1‐2* and *mur2‐2* seeds.
**Figure S6.** MALDI‐TOF mass spectra of *mur3‐3 xlt2‐1* compared to *mur3‐3* and *xlt2‐1* seeds.
**Figure S7.** Expression of XyG metabolism genes in developing seeds from pre‐globular to maturation green stage (http://www.bar.utoronto.ca/efp/cgi‐bin/efpWeb.cgi?dataSource=Seed).
**Figure S8.** XyG immunolocalization in wild type and *axy8*, *bgal10* and *xyl1* germinating seeds at endosperm rupture.
**Figure S9.** MS2 fragmentation pattern of *m*/*z* 1077 in negative mode.

**Table S1.** Gene specific primers used for expression analysis.

## Data Availability

All relevant data can be found within the manuscript and its supporting materials.
