# Supplementary material for: Analysis of xyloglucan metabolism mutants highlights the prominent role of xylose cleavage in seed dormancy
Source: Plant J. 2025 Mar 31;122(1):e70063. doi: 10.1111/tpj.70063 (PMC11956407; doi:10.1111/tpj.70063)
Supplement: Supplementary file 1 — Figure S1. Nomenclature and schematic representation of XyG showing enzymes involved in their synthesis and modification, as detailed in the text. Figure S2. Schematic diagram of XyG metabolism genes and mutants. Figure S3. Dormancy and ABA germination inhibition in mutant seeds compared to wild‐type (WT). Figure S4. MALDI‐TOF mass spectra of axy8‐1 bgal1‐2 xyl1‐2 compared to xyl1‐2 and wild type seeds. Figure S5. MALDI‐TOF mass spectra of mur1‐2 and mur2‐2 seeds. Figure S6. MALDI‐TOF mass spectra of mur3‐3 xlt2‐1 compared to mur3‐3 and xlt2‐1 seeds. Figure S7. Expression of XyG metabolism genes in developing seeds from pre‐globular to maturation green stage (http://www.bar.utoronto.ca/efp/cgi‐bin/efpWeb.cgi?dataSource=Seed). Figure S8. XyG immunolocalization in wild type and axy8, bgal10 and xyl1 germinating seeds at endosperm rupture. Figure S9. MS2 fragmentation pattern of m/z 1077 in negative mode. [file TPJ-122-0-s001.pdf]

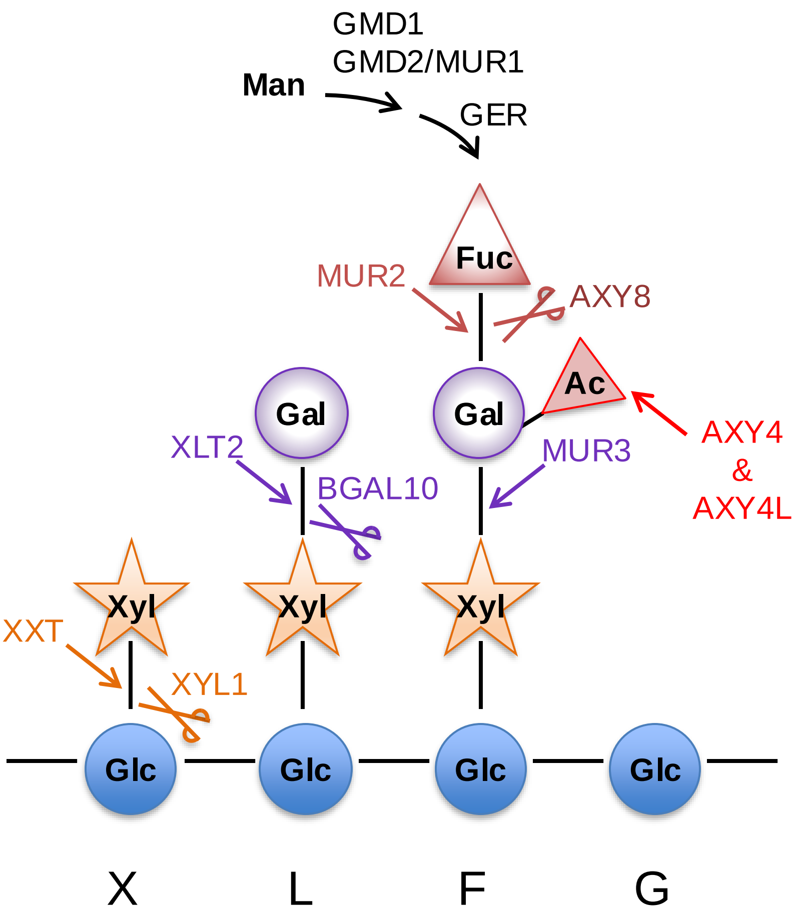

Figure S1. Nomenclature and schematic representation of XyG showing enzymes involved in their synthesis and modification, as detailed in the text. These oligosaccharide motifs are observed after endoglucanase cleavage at unsubstituted glucosyl residues. Glc, D-Glucose; Xyl, D-Xylose; Gal, D-Galactose; Fuc, L-Fucose; Ac, Acetyl.

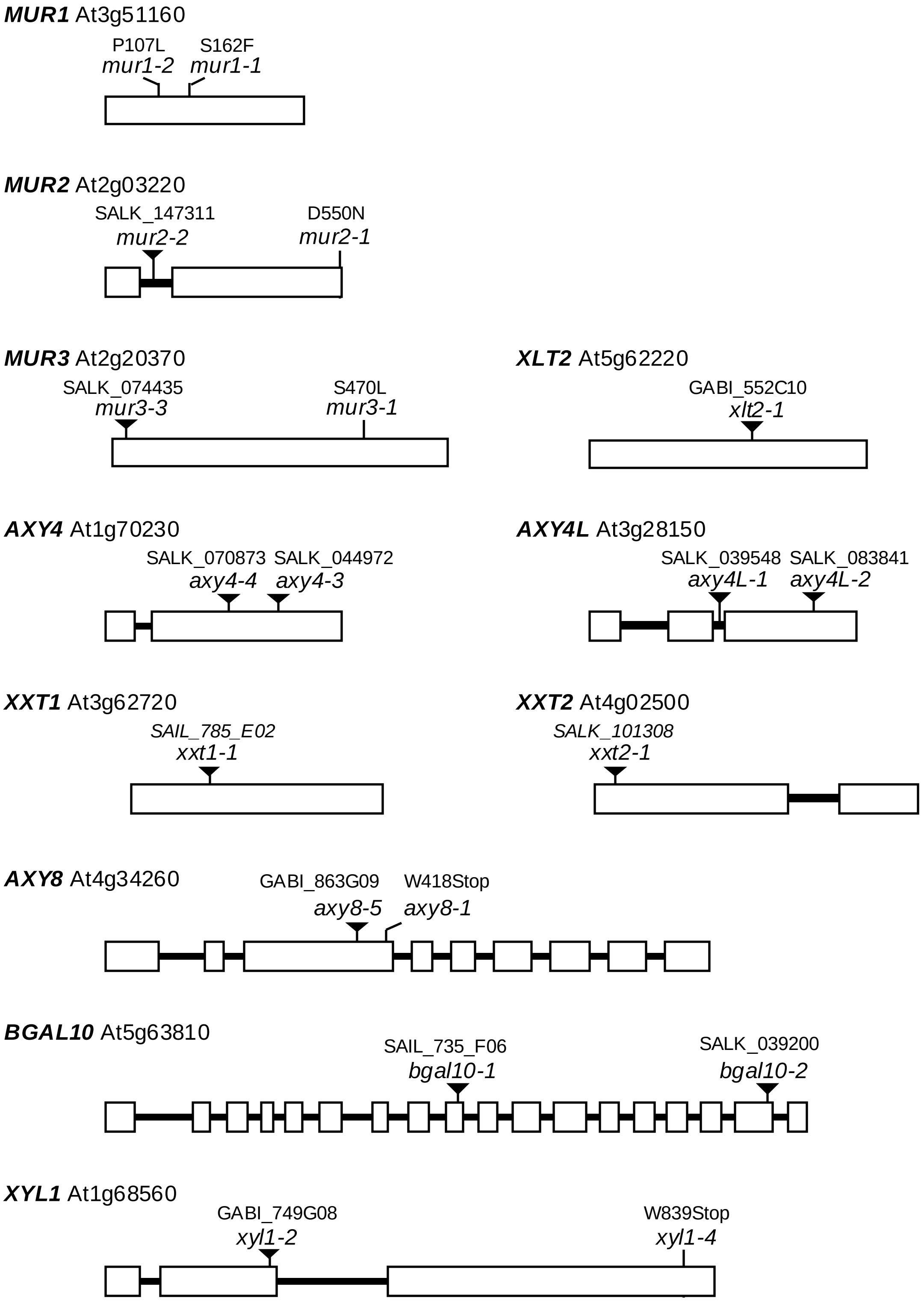

Figure S2. Schematic diagram of XyG metabolism genes and mutants.

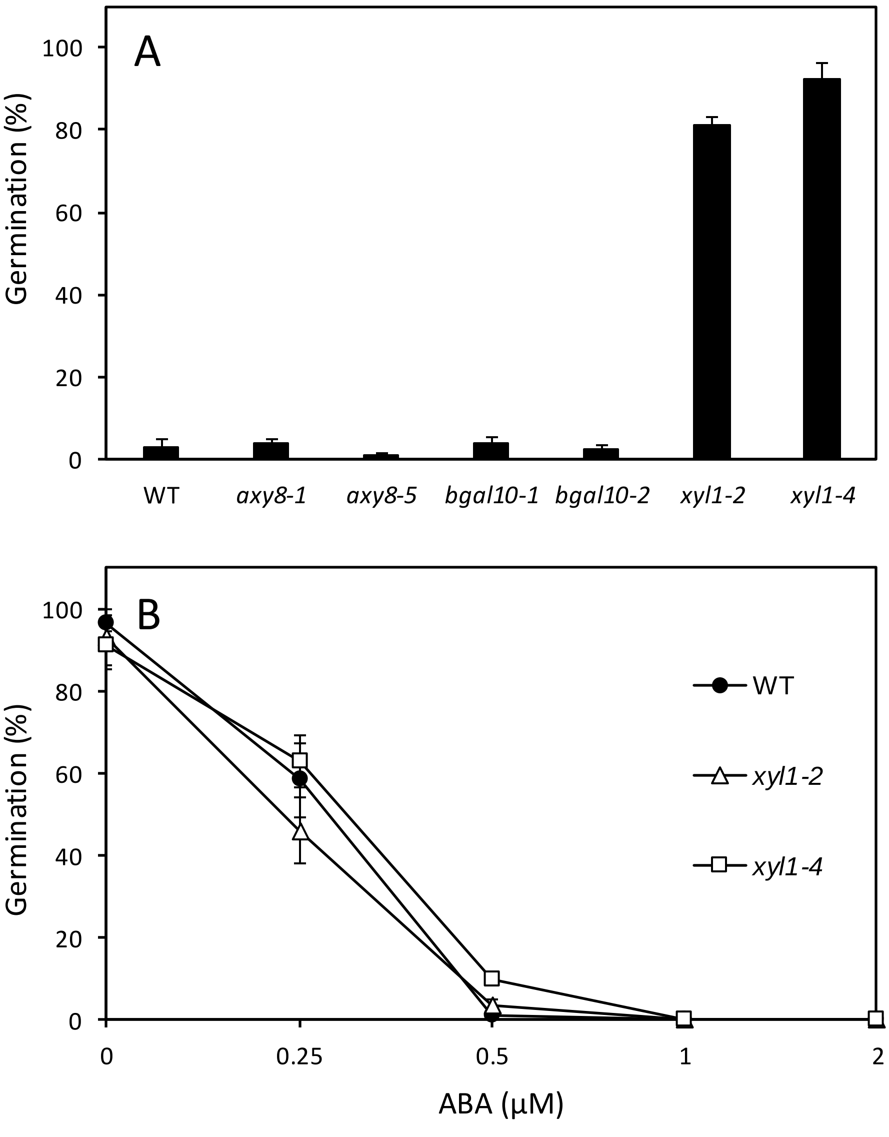

Figure S3. Seed dormancy and ABA inhibition of mutant seed germination compared to wild-type (WT). (A) Germination of freshly-harvested seeds of WT and mutant alleles, *axy8*, *bgal10* and *xyl1*. Germination (radicle protrusion) was scored 2 weeks after sowing. (B) Germination of stratified seeds of WT and *xyl1* alleles on media containing 0 to 4 µM ABA. Germination (green cotyledons) was scored one week after sowing. Means of three biological replicates are shown with SD.

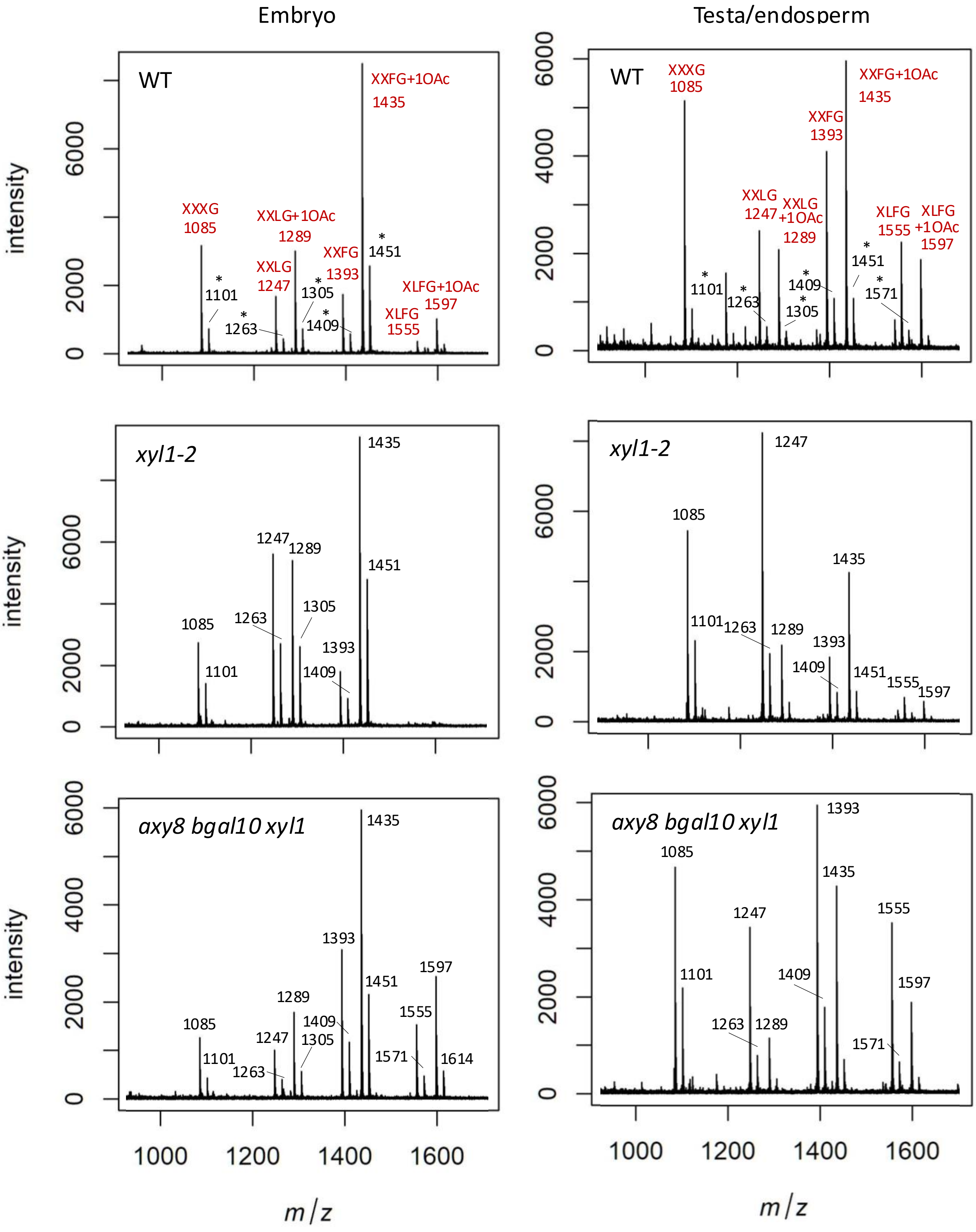

Figure S4. MALDI-TOF mass spectra of *axy8-1 bgal1-2 xyl1-2* compared to *xyl1-2* and wild type seeds. MALDI-TOF analysis was performed on dissected seeds after imbibition during 3 hours. Embryos were separated from testa and endosperm. The major peaks which relative abundance has been quantified (Fig. 2) are marked in red. Asterisks indicate non-quantified potassium adduct ions.

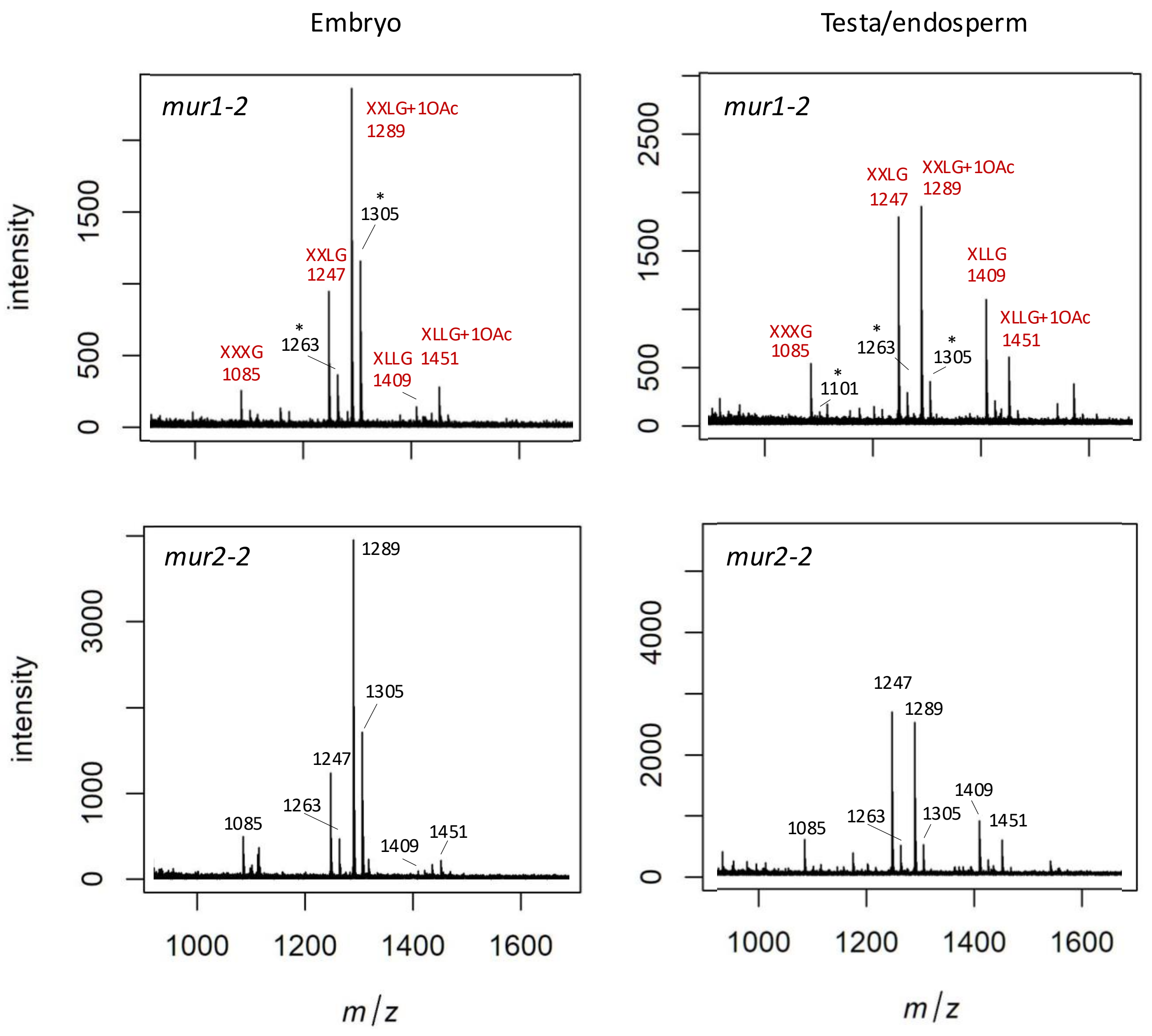

Figure S5. MALDI-TOF mass spectra of *mur1-2* and *mur2-2* seeds. MALDI-TOF analysis was performed on dissected seeds after imbibition during 3 hours. Embryos were separated from testa and endosperm. The major peaks which relative abundance has been quantified (Fig. 4) are marked in red. Asterisks indicate non-quantified potassium adduct ions.

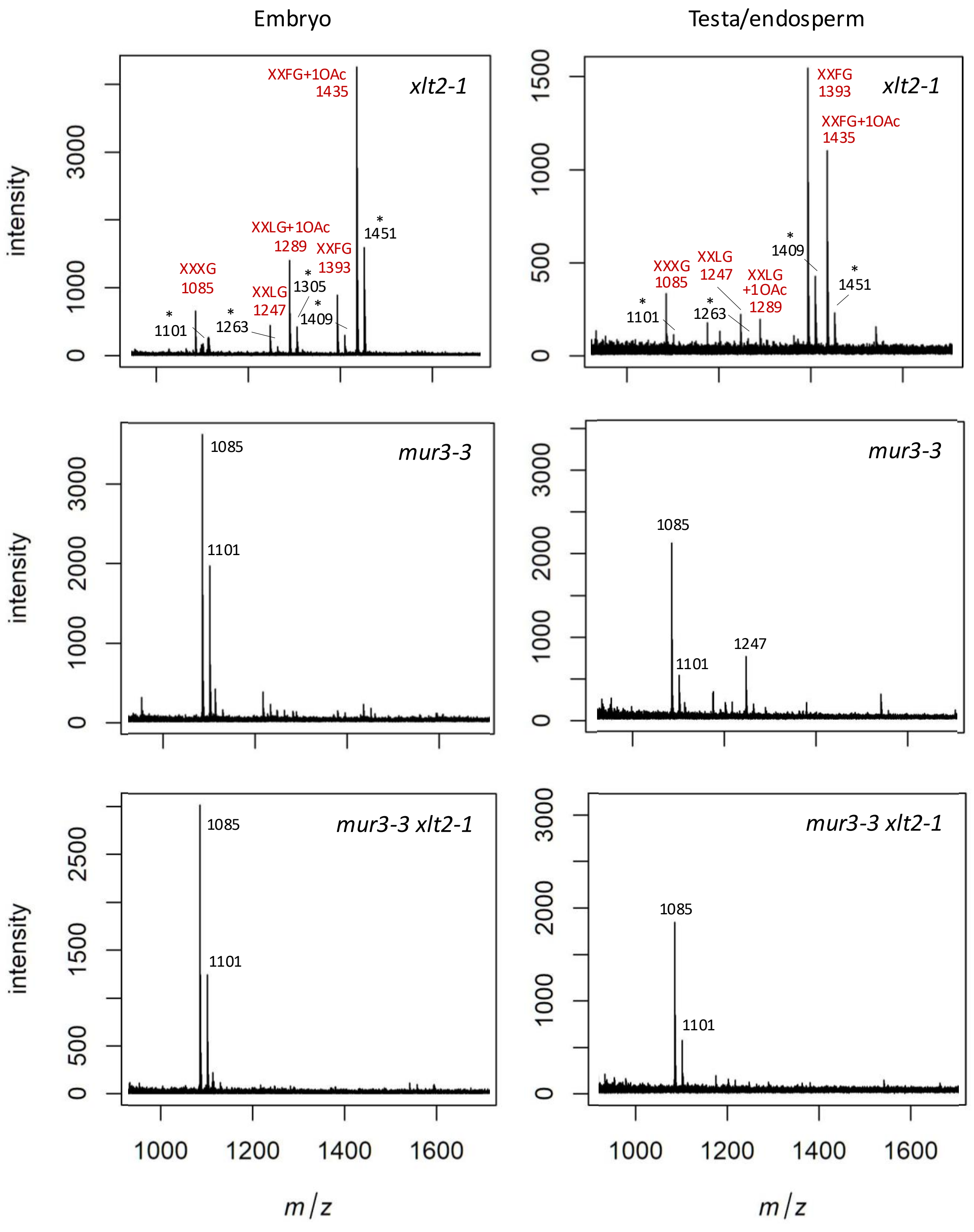

Figure S6. MALDI-TOF mass spectra of *mur3-3 xlt2-1* compared to *mur3-3* and *xlt2-1* seeds. MALDI-TOF analysis was performed on dissected seeds after imbibition during 3 hours. Embryos were separated from testa and endosperm. The major peaks which relative abundance has been quantified (Fig. 4) are marked in red. Asterisks indicate non-quantified potassium adduct ions.

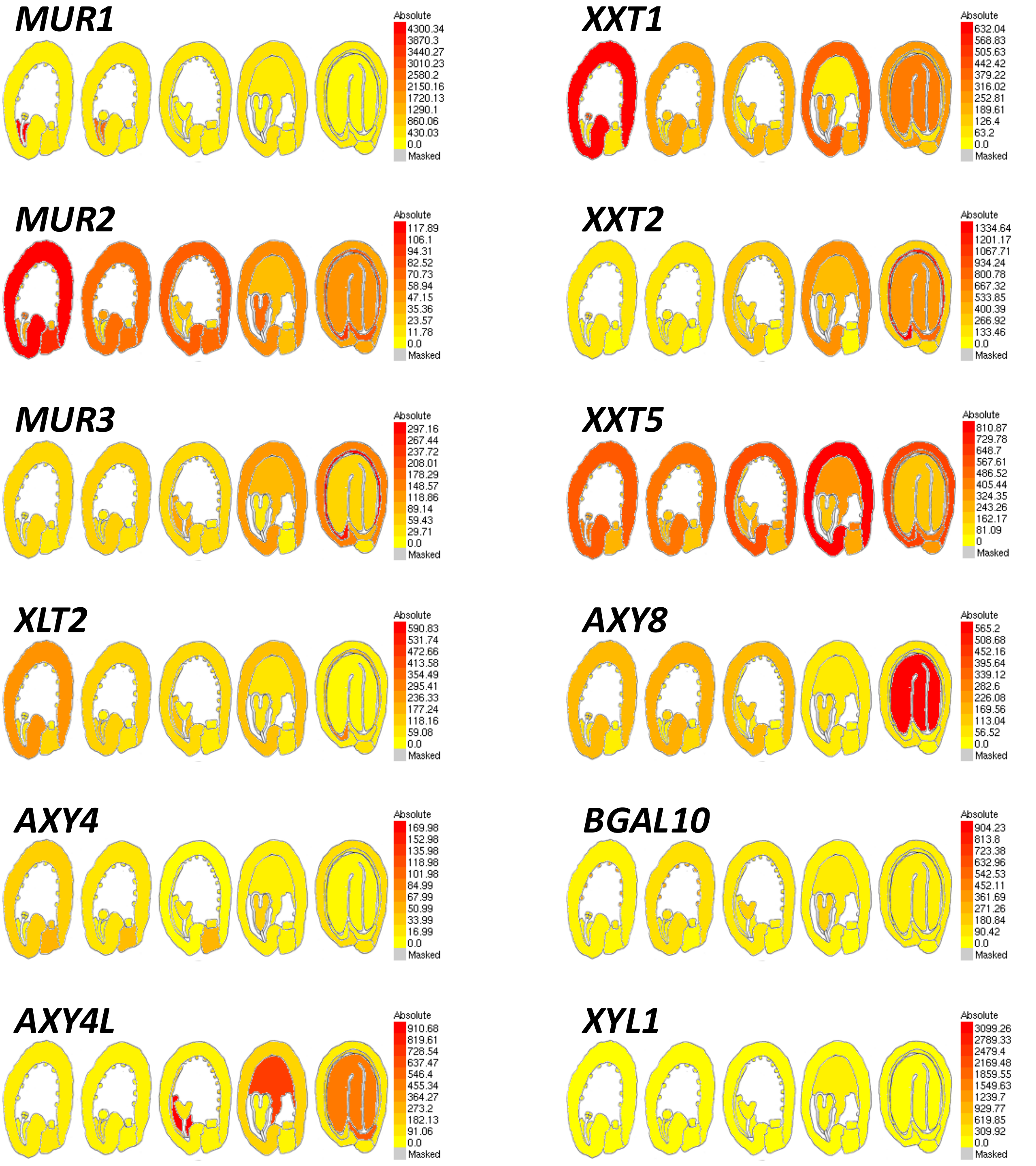

Figure S7. Expression of XyG metabolism genes in developing seeds from pre-globular to maturation green stage (http://www.bar.utoronto.ca/efp/cgi-bin/efpWeb.cgi?dataSource=Seed)

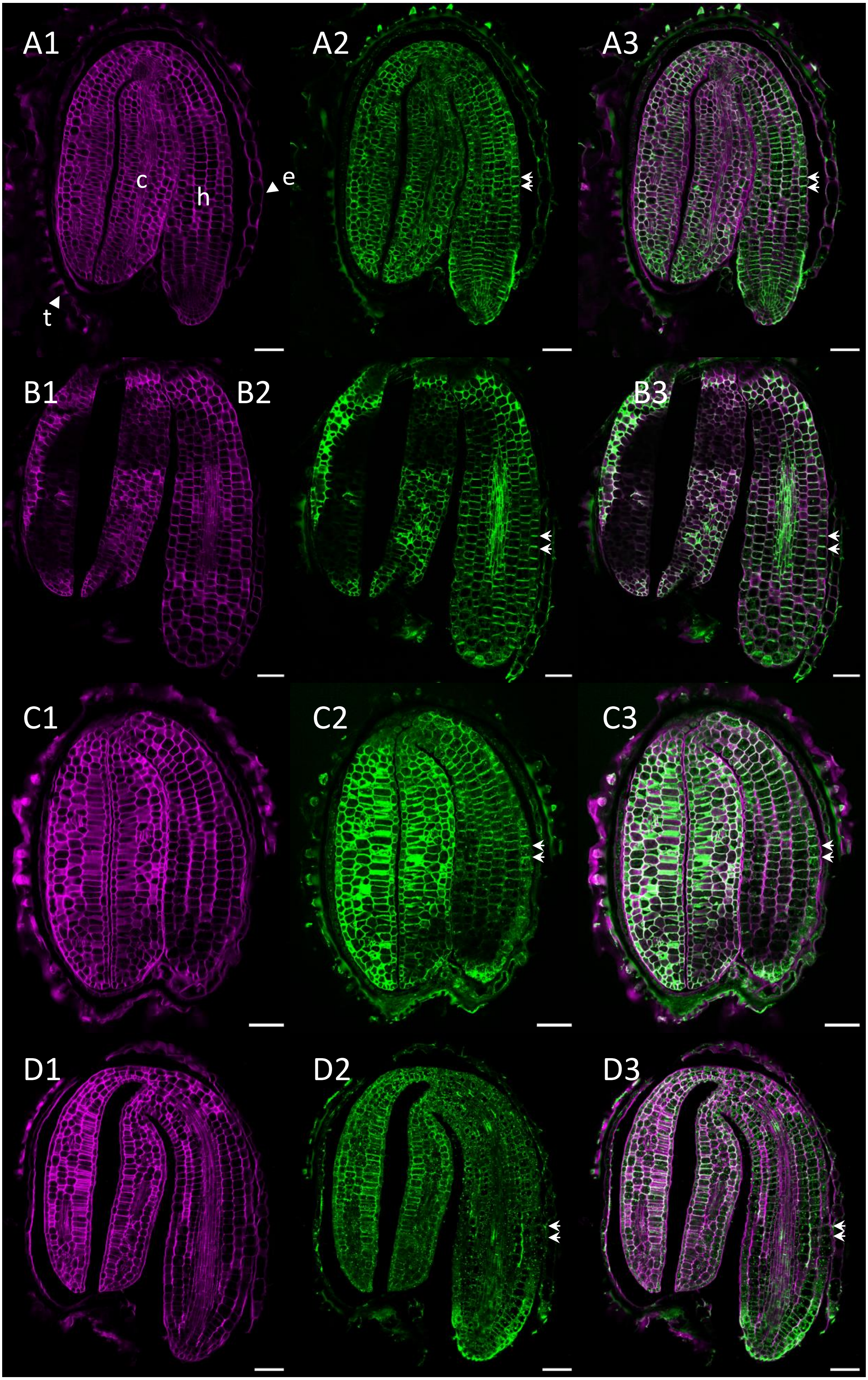

A1
A2
A3
c
h
e
t
B1
B2
B3
C1
C2
C3
D1
D2
D3

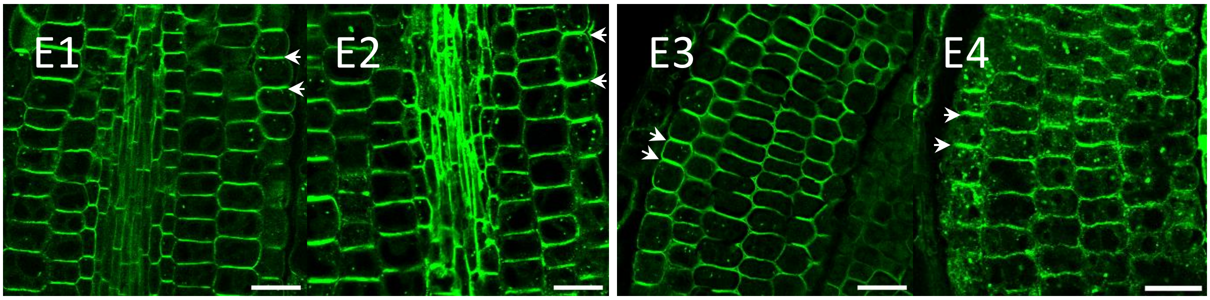

Figure S8. XyG mmunolocalisation in wild type and *axy8*, *bgal10* and *xyl1* germinating seeds at endosperm rupture. Whole seed sections of wild type (A), *axy8-1* (B), *bgal10-2* (C) and *xyl1-2* (D). In Magenta: cellulose labelling with calcofluor (A1-D1), in green: antibody labelling with CCRCM1 (A2-D2) and composite images showing both calcofluor and CCRCM1 labelling (A3-D3). Bar = 50 µm. Enlarged images of LM25 (E1, E3) and CCRCM1 (E2, E4) labelling in *axy8-1* (E1, E2) and *bgal10-2* (E3, E4) hypocotyls. Bar = 25 µm. c, cotyledons; h, hypocotyl; e, endosperm; t, testa. Double arrows highlight immunolabelling of transversal cell walls in embryo hypocotyl.

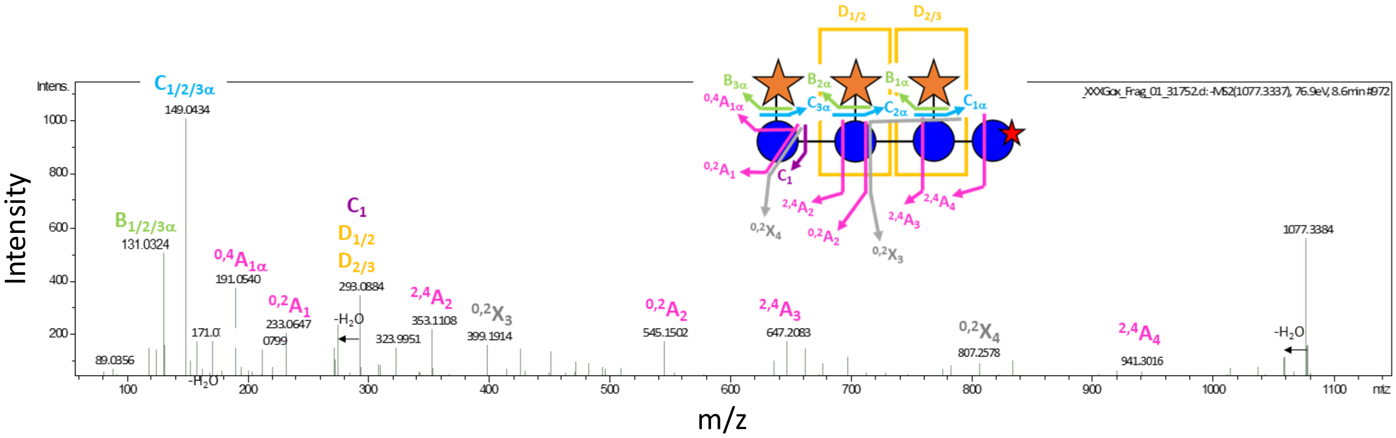

Figure S9. MS^2 fragmentation pattern of *m/z* 1077 in negative mode. The MS fragmentation pattern is annotated following the nomenclature of Domon & Costello (1988). Monosacharides are depicted using the Symbol Nomenclature For Glycans. The red star indicates oxidation.

Domon, B. & Costello, C. E. (1988) Systematic nomenclature for carbohydrate fragmentations in FAB-MS/MS spectra of glycoconjugates. *Glycoconjugate Journal*, **5**, 397–409. Available from: https://doi.org/10.1007/BF01049915
