# Supplementary material for: Analysis of xyloglucan metabolism mutants highlights the prominent role of xylose cleavage in seed dormancy
Source: Plant J. 2025 Mar 31;122(1):e70063. doi: 10.1111/tpj.70063 (PMC11956407; doi:10.1111/tpj.70063)
Supplement: Supplementary file 2 — Table S1. Gene specific primers used for expression analysis. [file TPJ-122-0-s002.pdf]

Table S1. Gene specific primers used for expression analysis

Gene	AGI		Primer sequence
	At4g12590	forward	TGGCATTGACTTGAGCACTGTCG
		reverse	TCGAGGTAGTGCCCATTCGTGCT
MUR1	At3g51160	forward	CACCCCAGATCTCCTTACGC
		reverse	CACGAAATTCTCACCACGGC
MUR2	At2g03220	forward	CGGATTATGGGTTTCGCCGAAG
		reverse	CCAGAAGCAAGTAGCCCTCC
MUR3	At2g20370	forward	GAGGAAGGCCAACGGGAAG
		reverse	GCATCTGCTGAAATAGTCGTGCC
XLT2	At5g62220	forward	CGCTGGACTCGCAGTTGG
		reverse	CCCATGTGATGCGTCCCATAG
AXY4	At1g70230	forward	GACACCTCTCTTCAAGACTCTGC
		reverse	CTGACCCAGTTCCCTTGCG
AXY4L	At3g28150	forward	GGTGATTCTGTGGCTAGGAACC
		reverse	CTTAGTCCACGAGGTCGAGAGAG
XXT1	At3g62720	forward	GGTGTTCGAGCTTCCATGGG
		reverse	GAAGATCAAGCGACCACTGTGAG
XXT2	At4g02500	forward	CTGTTGGCTACTCAGCGAGAC
		reverse	CCAATGGCCATCTGTGATCTCC
XXT5	At1g74380	forward	GGATTTGTTAGATGCTTGGGCG
		reverse	CAATGCTGACTGATCATCTGCCTC
AXY8	At4g34260	forward	CTCTTTGGCCTCTTCCCGG
		reverse	GACCATCCTGGTCCTTCCTCTC
BGAL10	At5g63810	forward	GGGTTGGAGCTGGACTTACAAG
		reverse	GATGCTCTCCTTCCACGCC
XYL1	At1g68560	forward	CGGAAGACCTGCTCCTATGC
		reverse	GCCACAGGATTCAACGTGAAATC
